# Harnessing anti‐tumor and tumor‐tropism functions of macrophages via nanotechnology for tumor immunotherapy

**DOI:** 10.1002/EXP.20210166

**Published:** 2022-02-25

**Authors:** Yanhui Zheng, Yaobao Han, Qiao Sun, Zhen Li

**Affiliations:** ^1^ Center for Molecular Imaging and Nuclear Medicine State Key Laboratory of Radiation Medicine and Protection School for Radiological and Interdisciplinary Sciences (RAD‐X) Collaborative Innovation Center of Radiation Medicine of Jiangsu Higher Education Institutions Soochow University Suzhou China

**Keywords:** immunotherapy, nanostructures, macrophage‐based drug carriers, tumor‐associated macrophages, tumor microenvironment

## Abstract

Reprogramming the immunosuppressive tumor microenvironment by modulating macrophages holds great promise in tumor immunotherapy. As a class of professional phagocytes and antigen‐presenting cells in the innate immune system, macrophages can not only directly engulf and clear tumor cells, but also play roles in presenting tumor‐specific antigen to initiate adaptive immunity. However, the tumor‐associated macrophages (TAMs) usually display tumor‐supportive M2 phenotype rather than anti‐tumor M1 phenotype. They can support tumor cells to escape immunological surveillance, aggravate tumor progression, and impede tumor‐specific T cell immunity. Although many TAMs‐modulating agents have shown great success in therapy of multiple tumors, they face enormous challenges including poor tumor accumulation and off‐target side effects. An alternative solution is the use of advanced nanostructures, which not only can deliver TAMs‐modulating agents to augment therapeutic efficacy, but also can directly serve as modulators of TAMs. Another important strategy is the exploitation of macrophages and macrophage‐derived components as tumor‐targeting delivery vehicles. Herein, we summarize the recent advances in targeting and engineering macrophages for tumor immunotherapy, including (1) direct and indirect effects of macrophages on the augmentation of immunotherapy and (2) strategies for engineering macrophage‐based drug carriers. The existing perspectives and challenges of macrophage‐based tumor immunotherapies are also highlighted.

## Graphical abstract

Modulating macrophages for tumor immunotherapy holds great promise to improve anti‐tumor efficacy. Inhibition of macrophage recruitment, depleting tumor‐associated macrophages (TAMs), repolarizing TAMs, and regulating macrophage‐mediated phagocytosis of tumor cells are the four major strategies for manipulating macrophage‐mediated tumor immunotherapy.

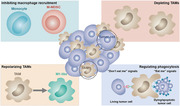

## 1 INTRODUCTION

Immune escape as a main feature of malignant tumors is controlled by multiple immunosuppressive cells, which include myeloid‐derived suppressor cells (MDSCs), tumor‐associated macrophages (TAMs), and regulatory T lymphocytes (Tregs) in the tumor microenvironment (TME).^[^ ^1^, ^2^, ^3^ ^]^ Among these cells, TAMs are the most abundant populations in the TME,^[^ ^4^, ^5^ ^]^ and are highly plastic. They can be classified into pro‐inflammatory or anti‐tumor M1‐like macrophages, and anti‐inflammatory or pro‐tumor M2‐like macrophages,^[^ ^6^ ^]^ of which M1‐like macrophages are much less than M2 phenotype within tumor. Therefore, TAMs are usually referred to the M2‐like macrophages within tumor. They are derived from macrophage precursors such as inflammatory monocytes and monocyte‐related myeloid‐derived suppressor cells (M‐MDSCs), which are recruited by abundant chemoattractants in the TME, subjected to immunosuppressive training, and then transformed into M2‐like TAMs to promote tumor cell migration, invasion, and metastasis.^[^ ^4^, ^7^, ^8^, ^9^ ^]^ M2‐like TAMs can also potently inhibit tumor‐specific T cell immunity through direct or indirect effects.^[^ ^8^ ^]^ For instance, they express inhibitory factors such as, CD206, arginase1 (Arg1), indoleamine 2,3‐dioxygenase (IDO) to directly inhibit T cell functions.^[^ ^8^, ^10^ ^]^ Correspondingly, CD206 can blunt CD8^+^ T cell cytotoxicity through inhibiting phosphatase activity of CD45.^[^ ^10^ ^]^ Arg1 and IDO can disrupt T cell cytotoxicity by inducing metabolic starvation of T cells.^[^ ^4^, ^11^ ^]^ TAMs also indirectly inhibit T cell activities by attracting immunosuppressive Treg cells. More importantly, as a subtype of professional antigen‐presenting cells (APCs), M2‐like macrophages usually display weak antigen presentation capability, while M1‐like macrophages up‐regulate major histocompatibility complex class II (MHC‐II) molecules and positive‐costimulatory molecules CD40, CD80, and CD86 to exhibit potent antigen presentation capability.^[^ ^6^, ^12^ ^]^ Therefore, developing strategies to decrease the number of M2‐like macrophages or promote M2‐to‐M1 repolarization holds great promise to bridge innate and adaptive immunity for enhancing tumor immunotherapies.

As one major component of the innate system, macrophages can severe as professional phagocytes to defense against malignancies. However, tumor cells can disguise themselves to bypass phagocytosis via expression of anti‐phagocytosis proteins.^[^ ^13^, ^14^ ^]^ In addition to phagocytosing living tumor cells at the tumor site, macrophages also swiftly engulf and clear dying/apoptotic cells (a process denoted as efferocytosis) to inhibit inflammatory response and facilitate the formation of M2‐like macrophages, thereby promoting tumor immune escape and progression.^[^ ^15^, ^16^, ^17^, ^18^, ^19^ ^]^ Since conventional anti‐tumor therapies such as chemotherapy, radiotherapy, and photodynamic therapy (PDT) can induce abundant tumor cell apoptosis, combination with suppression of macrophage‐mediated efferocytosis is beneficial to the further enhancement of their therapeutic efficacy.^[^ ^15^, ^20^ ^]^ Collectively, targeting phagocytosis checkpoints in the living tumor cells or inhibiting efferocytosis of apoptotic cells in the TME hold exciting potentials for improving anti‐tumor efficacy.

The recent application of nanotechnology in immunotherapy has attracted extensive attention. Nanosized particles can be modified to selectively accumulate at tumor site through passive/active targeting effects.^[^ ^3^ ^]^ The passive targeting can be achieved by modulating physical properties of nanoparticles such as, shape, size, and surface charge, etc.^[^ ^5^ ^]^ However, the passive targeting strongly depends on the enhanced permeability and retention (EPR) effect, which is poorly reproduced in human tumors.^[^ ^3^, ^5^ ^]^ Furthermore, the early stage tumors and postoperative minimal residual tumor lesions are EPR‐deficient.^[^ ^21^ ^]^ The active targeting can be realized by modifying nanoparticles with targeting ligands, such as, antibodies, peptides, or aptamers, which can selectively recognize specific receptors overexpressed on TAMs.^[^ ^3^, ^5^, ^22^ ^]^ Therefore, they have huge potential in directly regulating macrophage functions or indirectly delivering immunomodulators to reprogram TAMs, which can successfully avoid off‐target side effects and improve immunomodulatory efficacy.^[^ ^22^, ^23^ ^]^ Although active targeting can further enhance macrophage‐targeting, the targeting ligands on the surface of nanoparticles can induce a “binding site barrier” due to the strong ligand‐receptor interactions, leading to limited tumor penetration of nanoparticles.^[^ ^5^ ^]^ More importantly, the highly dynamic TME consists of multiple biological barriers including dense extracellular matrix (ECM), high interstitial fluid pressure (IFP), heterogeneous blood supply, tumor stroma, etc., which are challenges for nanoparticles penetrating into the deep tumor site.^[^ ^24^, ^25^, ^26^, ^27^ ^]^

An alternative strategy is to engineer macrophages or macrophage‐derived components (including macrophage membrane and macrophage‐derived extracellular vesicles (EVs)), because macrophages have natural tumor‐homing capability, extravasate through the tight vascular wall, and arrive at the hypoxic area or poorly vascularized region.^[^ ^21^, ^28^, ^29^ ^]^ They can also exhibit excellent stealth capability of evading the phagocytosis of mononuclear phagocyte system (MPS), leading to much long blood circulation.^[^ ^21^ ^]^ It should be noted that the macrophage‐derived components inherit these properties and possess the capability of tumor tropism.^[^ ^30^, ^31^, ^32^, ^33^ ^]^ Therefore, these macrophages‐based strategies hold great promise to improve tumor immunotherapies via overcoming complex biological barriers for deep penetrable tumor‐targeted drug delivery.

**FIGURE 1:**
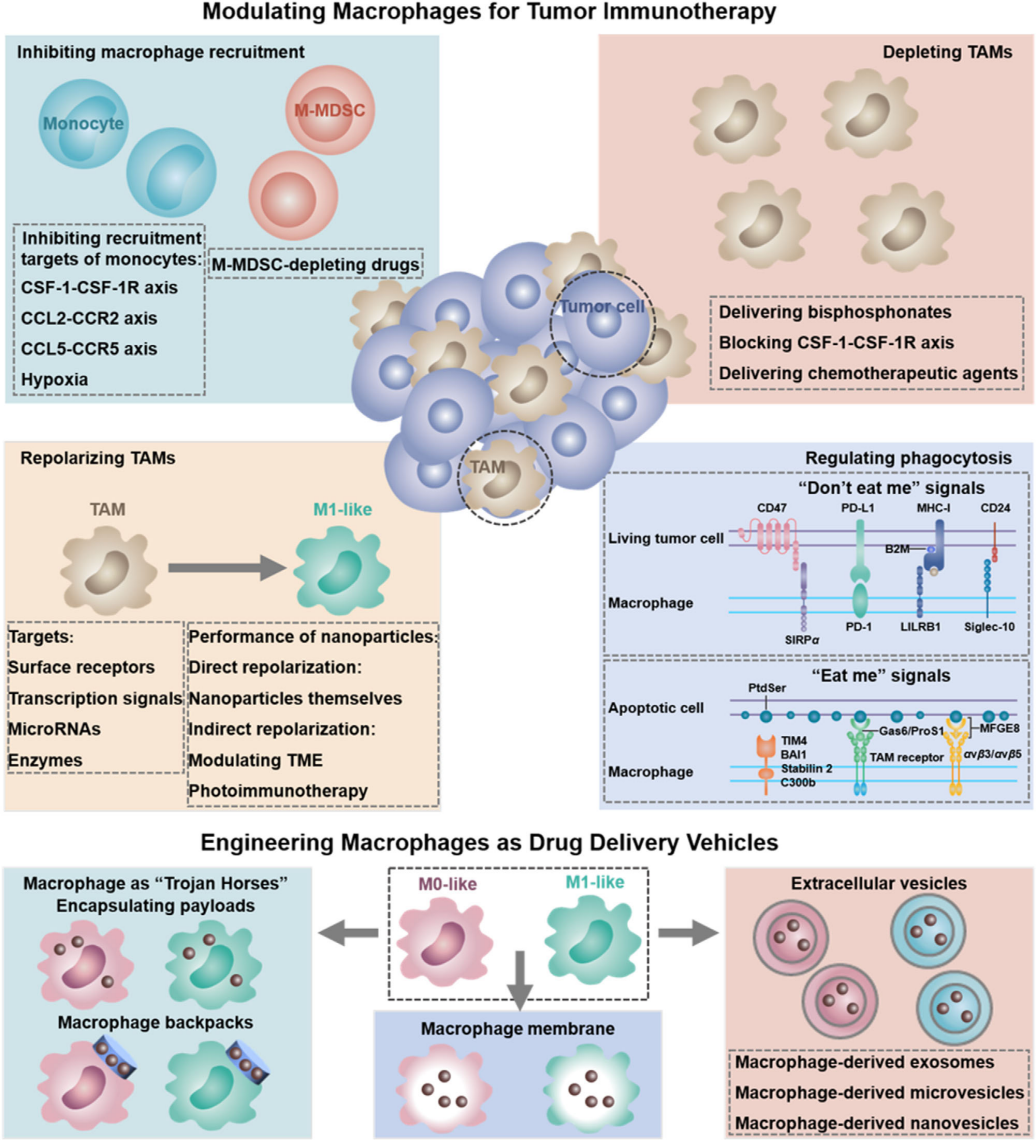
Modulating macrophages for tumor immunotherapy and engineering macrophages as drug delivery vehicles. Inhibition of macrophage recruitment, depleting TAMs, repolarizing TAMs, and regulating macrophage‐mediated phagocytosis of tumor cells are the four major strategies for manipulating macrophage‐mediated tumor immunotherapy. Engineering macrophages as drug delivery carriers is very promising for tumor immunotherapy, including engineering macrophages as “Trojan Horses,” utilizing macrophage‐derived components, such as, macrophage membrane, macrophage‐derived extracellular vesicles (i.e., exosomes, microvesicles, or nanovesicles), for tumor‐targeting delivery of anti‐tumor payloads

In summary, TAMs in the TME originate from circulating macrophage precursors such as, inflammatory monocytes and M­‐MDSCs, which are susceptible to immunosuppressive training, and then differentiate into tumor‐supportive M2‐like TAMs.^[^ ^4^, ^34^, ^35^ ^]^ The macrophages are dynamically changeable and can be converted into anti‐tumor M1 phenotype or tumor‐supportive M2 phenotype in response to different stimuli.^[^ ^4^, ^35^ ^]^ Additionally, macrophages are naturally professional phagocytes with tumor‐phagocytosis capability.^[^ ^13^, ^17^ ^]^ In this review, we systematically summarize the recent advances in macrophage‐based tumor immunotherapies by using four strategies (Figure 1), including (1) elimination of TAMs sources, (2) direct depletion of the existing TAMs, (3) repolarization of TAMs, and (4) modulation of macrophage‐mediated tumor‐phagocytosis. The classification was initially proposed by Xia et al.^[^ ^36^ ^]^ Then, we highlight the advances of macrophage‐based drug delivery system based on their natural tumor‐homing capability.^[^ ^5^, ^28^, ^30^ ^]^ Finally, we provide perspectives and challenges in harnessing macrophage‐based immunotherapies for improving the anti‐tumor response.

## 2 MACROPHAGE AND TUMOR‐ASSOCIATED MACROPHAGES ORIGINS

### 2.1 Origins of macrophages

As the most important immune cells in the innate immune system, macrophages are found throughout the body's tissues to maintain organism's homeostasis by responding to the physiological changes and external stimuli.^[^ ^37^ ^]^ They are mainly developed from three sources, including yolk sac, fetal liver, and bone marrow. (1) The F4/80^hi^ tissue‐resident macrophages are considered from embryonic precursors in fetal yolk sac during the development of embryo. These precursors spread throughout tissues (i.e., brain, pancreas, spleen, liver, lung, and kidney) where they differentiate into corresponding matured tissue‐resident macrophages, and exert their functions to regulate tissue homeostasis.^[^ ^5^, ^12^, ^38^ ^]^ (2) Langerhans cells are derived from the fetal liver progenitors. (3) The F4/80^low^ macrophages are derived from Ly6c^+^ monocytes, which are originated from the bone marrow.^[^ ^12^ ^]^ It is worthy noted that the inflammatory Ly6c^+^ monocytes are usually recruited into sites with injury, infection, or inflammation and matured into macrophages, while the Ly6c^–^ patrolling monocytes exert functions to monitor intravascular pathogens and protect lung microvasculature. They rarely migrate to tissues to transform into macrophages.^[^ ^4^, ^12^, ^39^ ^]^

### 2.2 Origins and roles of tumor‐associated macrophages

**FIGURE 2:**
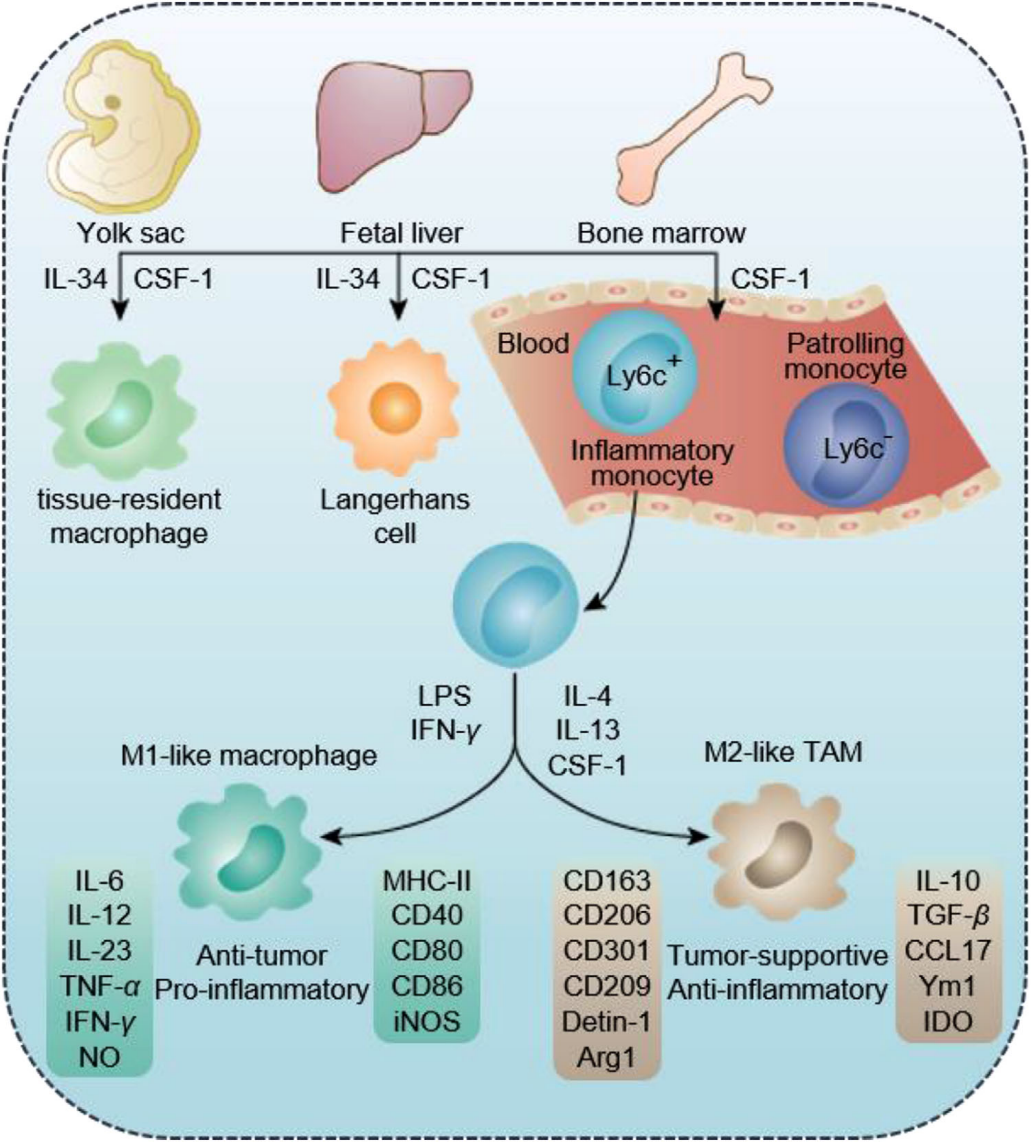
Origins of macrophages and TAMs. Macrophages in the body's tissues are mainly originated from yolk sac, fetal liver, and bone marrow, of which the bone‐marrow‐derived circulating inflammatory Ly6c^+^ monocytes are the main source of tumor‐supportive M2‐like TAMs. Monocytes can differentiate into M1‐ or M2 like macrophages in response to different stimuli. LPS or IFN‐*γ* usually induces them differentiating into anti‐tumor M1 phenotype macrophages, which secrete IL‐6, IL‐12, IL‐23, TNF‐*α*, IFN‐*γ*, and NO, and overexpress MHC‐II, positive‐costimulatory molecules (e.g., CD40, CD80, and CD86) and iNOS. IL‐4, IL‐13, or CSF‐1 can promote them polarizing into M2 phenotype macrophages, which secrete IL‐10, TGF‐*β*, CCL17, Ym1, and IDO, and overexpress haemoglobin scavenger receptor CD163, C‐type lectin receptors (CD206, CD301, CD209, and detin‐1), as well as Arg1

Previous reports have demonstrated that TAMs are mainly originated from the bone‐marrow‐derived circulating inflammatory monocytes, which are recruited to the TME in response to inflammatory factors generated by tumor cells at the primary and metastatic sites, and differentiated into tumor‐supportive TAMs (Figure 2).^[^ ^34^, ^40^, ^41^ ^]^ The CC‐chemokine ligand 2 (CCL2) can recruit inflammatory monocytes, which can differentiate into TAMs (i.e., denoted as TAMs in the primary tumor, and metastasis‐associated macrophages (MAMs) in the metastasis tumor) by interaction with CCR2.^[^ ^34^, ^37^, ^40^, ^42^, ^43^ ^]^ Moreover, CCL2 can also extend the retention of MAMs by promoting their secretion of CCL3 at metastatic sites.^[^ ^44^, ^45^ ^]^ However, only a few types of tumors have different origins of tumor‐supportive TAMs. For example, the macrophages are a mixture of embryonic‐derived tissue‐resident populations and circulating inflammatory monocytes in the glioma and pancreatic cancer.^[^ ^46^, ^47^ ^]^ In the glioma, tumor progression and the poor prognosis were mainly ascribe to the infiltrating Ly6c^+^ inflammatory monocytes rather the resident microglia.^[^ ^46^ ^]^ However, in the pancreatic ductal adenocarcinoma (PDAC), the tumor‐supportive TAMs were replenished by embryonic‐derived tissue‐resident macrophages rather than bone‐marrow‐derived circulating inflammatory monocytes.^[^ ^47^ ^]^ Whatever the sources of these precursors, they can differentiate into new tumor‐supportive phenotypes.^[^ ^34^ ^]^

In addition to bone‐marrow‐derived circulating inflammatory monocytes, the circulating M‐MDSCs are also available to rapidly differentiate into TAMs.^[^ ^7^, ^48^ ^]^ Therefore, the different origin cues of TAMs suggest that targeting specific subpopulations of these cells for different types of tumors is needed for individual TAMs‐based tumor immunotherapy. Whatever the sources of macrophages, the tumor‐promoting influence of TAMs is strongly depended on the concentrations and roles of monocyte attractants and cytokines, which facilitate macrophages polarizing into tumor‐supportive TAMs in the TME.^[^ ^35^, ^43^ ^]^ The colony‐stimulating factor 1 receptor (CSF‐1R, a transmembrane tyrosine kinase class III receptor) is a key regulator for controlling differentiation, proliferation and survival of macrophages.^[^ ^12^, ^34^ ^]^ It has two ligands CSF‐1 and interleukin‐34 (IL‐34) to exert different functions, depending on the macrophage origin. The CSF‐1 mainly regulates the differentiation of yolk sac‐derived and bone‐marrow‐derived macrophages,^[^ ^43^ ^]^ while IL‐34 prefers to regulate the development of microglia and Langerhans cells.^[^ ^34^, ^37^, ^49^ ^]^ Therefore, CSF‐1 is an important attractant for monocytes recruitment.^[^ ^34^, ^35^, ^43^ ^]^ In addition, the previously mentioned CCL2 is also a crucial factor for monocyte recruitment.^[^ ^43^ ^]^ After being recruited into the tumor site, many factors can facilitate monocytes to differentiate into tumor‐supportive M2 phenotype to promote tumor progression.^[^ ^43^ ^]^

As mentioned previously, macrophages are divided into anti‐tumor M1 pro‐inflammatory type and tumor‐supportive M2 anti‐inflammatory type. The interferon‐*γ* (IFN‐*γ*) secreted by CD8^+^ T cells, natural killer (NK) cells, and other T helper 1 (Th1) cells can induce the formation of M1‐like macrophages. In addition, the lipopolysaccharide (LPS) can also promote the formation of M1‐like macrophages.^[^ ^6^, ^35^ ^]^ The M1‐like macrophages can up‐regulate pro‐inflammatory cytokines and markers including IL‐6, IL‐12, IL‐23, tumor necrosis factor (TNF)‐*α* (TNF‐*α*), IFN‐*γ*, inducible nitric oxide synthase (iNOS) and NO, and enhance their antigen presentation capability with increased MHC‐II and positive‐costimulatory molecules (e.g., CD40, CD80, and CD86).^[^ ^12^, ^50^, ^51^ ^]^ In the early stage of tumor initiation, the TME is dominated by a Th1 pro‐inflammatory circumstance, which benefits to the retention of anti‐tumor M1‐like macrophages.^[^ ^36^, ^43^ ^]^ Once the tumor is formed, the TME would turn into Th2 type circumstance, which is dominated by many immunosuppressive factors including IL‐4, IL‐13, CSF‐1, prostaglandins, and lactic acid, as well as, hypoxia condition, etc.^[^ ^23^, ^43^, ^51^, ^52^ ^]^ This abnormal TME can facilitate macrophages polarizing toward tumor‐supportive M2‐like TAMs, which exhibit decreased antigen presentation capability and express immunosuppressive factors including haemoglobin scavenger receptor CD163 as well as C‐type lectin receptors (CD206, CD301, CD209, and detin‐1), Arg1, IL‐10, TGF‐*β*, CCL17, Ym1, and IDO, etc.^[^ ^4^, ^12^, ^23^, ^50^, ^53^ ^]^ TAMs can facilitate tumor progression, invasion and intravasation, metastasis, angiogenesis, and suppress tumor‐specific T cell immunity.^[^ ^8^, ^12^ ^]^ In addition, they can also express matrix metalloproteinases (MMPs) such as, MMP9 and cathepsins to remodel the matrix for tumor intravasation.^[^ ^22^, ^37^ ^]^ Considering the important tumor‐killing and tumor‐promoting roles of macrophages, they could be a perfect target and tool for tumor treatment.

## 3 MODULATING MACROPHAGES FOR TUMOR IMMUNOTHERAPY

According to the sources of TAMs and the functions of different phenotypes, macrophage‐based tumor immunotherapy can be classified into four categories: (1) Inhibiting monocyte or M‐MDSC migration into tumor, (2) depleting TAMs, (3) repolarizing TAMs, and (4) regulating macrophage‐mediated tumor phagocytosis. Due to the traditional TAMs‐modulators face the challenges of nonspecific targeting, limited drugs delivery efficiency, rapid blood clearance, and system toxicity, nanosized particles are rationally designed to deliver them or serve as TAMs‐regulators, because they can be fabricated into different shapes with tunable size, surface charge, and targeting ligands to selectively accumulate at tumor site through passive/active targeting effects.^[^ ^3^, ^5^, ^54^, ^55^ ^]^ Furthermore, nanoparticles can be internalized by the intrinsic phagocytosis capability of macrophages, which is beneficial for the efficient accumulation of nanoparticles and their payloads in the tumor to improve their tumor penetration.^[^ ^55^ ^]^ More importantly, stimuli‐activatable nanomedicines show controllable drug release profile.^[^ ^26^ ^]^ Therefore, engineering nanoparticles for tumor‐targeted delivery of TAMs‐modulators or for directly regulating TAMs holds great potential to improve tumor‐specific accumulation, blood circulation time of modulators, and thus reduce adverse effects, which can enhance TAMs‐modulating efficacy.^[^ ^23^, ^54^ ^]^ Herein, we will focus on how to utilize nanoparticles or nanomedicines to improve the therapeutic efficacy of four strategies mentioned above.

### 3.1 Inhibiting the migration of circulating monocyte/macrophages/M‐myeloid‐derived suppressor cell into tumor

One of the most well‐established strategies for modulating TAMs is to inhibit the migration of monocytes (the main supplier of TAMs) into tumor. Since CSF‐1R is exclusively expressed by monocytic lineage cells (such as, monocytes and macrophages), it's ligand CSF‐1 secreted by many types of tumors is a key regulator for macrophage differentiation, proliferation, survival, and maturation.^[^ ^4^, ^34^, ^35^, ^56^ ^]^ Therefore, blocking CSF‐1‐CSF‐1R axis could inhibit the migration of TAMs precursor monocytes. The inhibitors of this axis are extensively tested in both preclinical and clinical studies, including antibodies, small molecule inhibitors, and siRNAs, etc.^[^ ^22^, ^36^ ^]^ CCL2 is also a potent attractant to recruit monocytes into primary and metastatic tumor sites. Inhibiting CCL2‐CCR2 has shown benefits to inhibition of tumor growth in many types of tumor models including prostate, mammary carcinoma, lung cancer, hepatocellular cancer, liver cancer, PDAC, and melanoma.^[^ ^34^, ^57^, ^58^, ^59^, ^60^, ^61^, ^62^ ^]^ However, discontinuing anti‐CCL2 treatment would trigger recruitment of monocytes again and thus aggravate lung metastasis in mouse breast tumor.^[^ ^8^, ^34^ ^]^ Additionally, inhibition of CCL5‐CCR5 axis is also an attractive solution to inhibit macrophage recruitment and suppress tumor progression in the breast tumor.^[^ ^44^ ^]^

**FIGURE 3:**
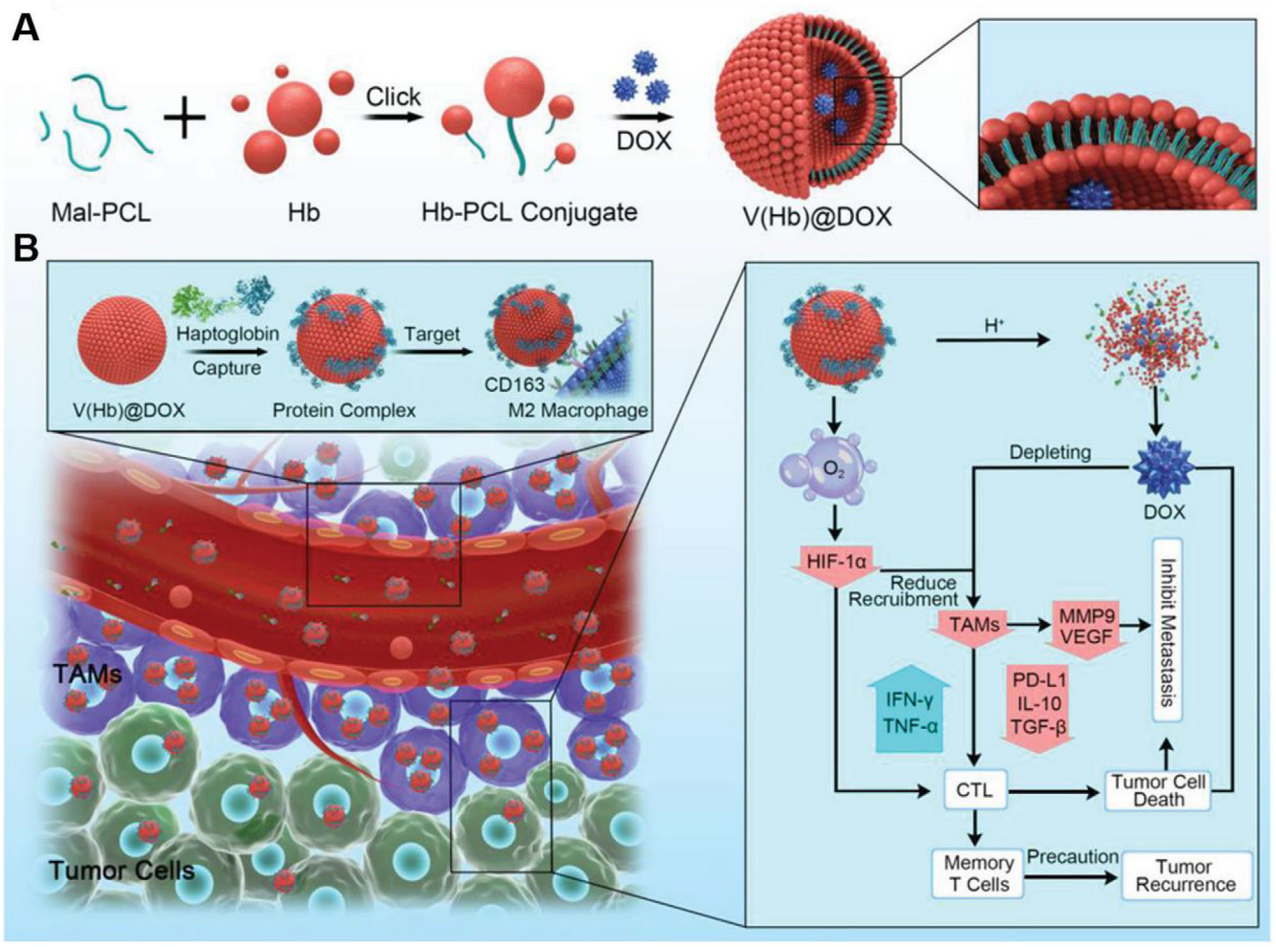
Schematic illustration of preparing nano‐RBC‐based TAMs‐targeting system for reversing immunosuppressive TME to improve chemoimmunotherapy. (A) Preparation of the DOX‐encapsulated biomimetic nano‐RBC system (V(Hb)@DOX). (B) Inhibition of TAMs‐recruitment improved chemo‐immunotherapy. Reproduced with permission.^[^ ^63^ ^]^ Copyright 2021, John Wiley & Sons

In addition to inhibition of CSF‐1‐CSF‐1R, CCL2‐CCR2, and CCL5‐CCR5 axes, amelioration of tumor hypoxia can also reduce the recruitment of macrophages. Wang et al. fabricated a self‐assembled biomimetic nano red blood cell system from hemoglobin‐poly(ε‐caprolactone) and chemotherapeutic doxorubicin (DOX) (V(Hb)@DOX) (Figure 3).^[^ ^63^ ^]^ The Hb moiety can interact with the endogenous plasma haptoglobin (Hp), which can selectively target the M2‐like macrophages through surface CD163 protein and then deliver oxygen (O_2_) to the TME. By alleviating the hypoxia, the capability of tumor recruiting M2‐like TAMs has been reduced, which is beneficial to the improvement of immunosuppressive TME.

As aforementioned, the circulating M‐MDSC is also an important source of tumor‐supportive TAMs because they are susceptible to the stimuli and rapidly differentiate into TAMs.^[^ ^7^, ^48^ ^]^ Therefore, depletion of M‐MDSC may hold great potential to reverse their adverse effects. Gemcitabine (Gem) is a popular MDSC‐depleting drug. Similar to Gem, lipid‐coated calcium phosphate and gemcitabine monophosphate nanoparticles could efficiently deplete tumor‐infiltrating M‐MDSCs and thus decrease tumor‐supportive M2‐like TAM populations, as well as, promote M2‐to‐M1 repolarization.^[^ ^48^ ^]^

### 3.2 Depleting tumor‐associated macrophages

Bisphosphonates are cytotoxic to myeloid cells and can be phagocyted by osteoclasts (bone macrophages), they are traditionally used for treating osteoporosis and preventing bone metastasis.^[^ ^4^, ^64^, ^65^, ^66^ ^]^ In addition to bone macrophages, they can be also internalized by TAMs to affect their functions.^[^ ^67^ ^]^ In particular, since liposomes can be heavily phagocytized by macrophages, the clodronate‐encapsulated liposomes are widely utilized for TAMs depletion.^[^ ^8^, ^68^, ^69^, ^70^, ^71^, ^72^ ^]^ Besides, zoledronic acid, the other type of bisphosphonates containing nitrogen can also reduce TAMs populations in different tumors.^[^ ^73^, ^74^, ^75^ ^]^ Huang et al. fabricated coordination polymer nanorods based on gadolinium and zoledronic acid that could self‐assemble in the TME.^[^ ^76^ ^]^ The resultant ZGd‐NRs could deposit X‐rays to generate reactive oxygen species (ROS) and induce immunogenic cell death (ICD). The released zoledronic acid could significantly deplete the TAMs, thereby alleviating immunosuppressive TME. Combining with these two merits, this nanorods elicited potent immune activation to inhibit breast tumor progression.

As mentioned previously, CSF‐1R is restrictively expressed by the macrophages and monocytes. CSF‐1‐CSF‐1R axis is important for TAMs survival, it can also serve as a target for directly interfering with TAMs for depletion.^[^ ^4^, ^35^, ^56^ ^]^ Serval small molecules and antibodies with capability of targeting CSF‐1‐CSF‐1R signaling are undergoing clinical trials. For example, in preclinical studies of glioblastoma, both PLX3397 and pexidartinib were effective for reducing TAMs and resulted in an effective anti‐tumor response.^[^ ^34^, ^77^, ^78^ ^]^ Qian et al. fabricated a type of dual‐targeting nanoparticles by linking a biocompatible M2‐targeting peptide with anti‐CSF‐1R siRNA.^[^ ^56^ ^]^ These nanoparticles could selectively block CSF‐1‐CSF‐1R signaling of M2‐like TAMs and deplete them from the TME, resulting in a decreased tumor size (87%) and prolonged survival time of melanoma tumor‐bearing mice.

Chemotherapeutic agents such as, trabectedin, epirubicin, DOX, and dasatinib have also been found to show capability of depleting TAMs.^[^ ^4^, ^36^, ^79^ ^]^ For instance, trabectedin can elicit specific cytotoxicity of circulating monocytes and TAMs via activating the TNF‐related apoptosis‐inducing ligand (TRAIL)‐caspase8 signaling pathway.^[^ ^4^, ^34^ ^]^ It has been demonstrated that monocytes and macrophages can specifically sense TRAIL due to their expression of TRAIL receptors (i.e., TRAILR1 and TRAILR2), making TRAILRs become an attractive target for depleting TAMs.^[^ ^80^ ^]^ More interestingly, trabectedin can also deplete another TAMs source M‐MDSCs, which is indeed an attractive ablation agent of TAMs.^[^ ^4^ ^]^ Liu et al. developed matrix MMP2‐sensitive phosphatidylserine (PtdSer) modified nanoparticles, which were loaded with the anti‐tumor drug dasatinib as a TAMs‐depleting agent.^[^ ^79^ ^]^ After the nanoparticles were accumulated in the MMP2 overexpressed tumor environment, the PtdSer was inversed from inside to outside to be specifically phagocytosed by TAMs, because externalization of PtdSer on the apoptotic cell membrane is a well‐known “eat me” signal to attract macrophages recognizing and phagocytosing apoptotic cells. This strategy could remarkably enhance the TAMs‐specific phagocytosis and effectively deplete TAMs, resulting in excellent anti‐tumor effect.

### 3.3 Repolarizing tumor‐associated macrophages

Inhibiting migration of monocytes and macrophages and depleting macrophages would reduce the populations of all macrophages including tissue‐resident macrophages, which can impair their functions in keeping homeostasis.^[^ ^8^, ^35^ ^]^ More importantly, indiscriminate ablation of all macrophages could also weak their intrinsic capability of tumor‐phagocytosis and tumor‐specific antigen presentation, because they are a major class of professional phagocytes and professional APCs.^[^ ^13^, ^35^ ^]^ Since M1‐like macrophages display anti‐tumor effect with potent antigen presentation ability, reprogramming M2‐like TAMs into anti‐tumor M1 phenotype is the popular strategy for macrophage‐mediated tumor immunotherapy in these years.

The current strategies of repolarizing macrophages are mainly focused on the following aspects: (1) Targeting TAMs surface receptors (CD206 and CD301, toll‐like receptors (TLRs), CSF‐1R, MARCO, CD40, etc.), (2) modulating transcription signals (activator of transcription 1 (STAT1), STAT5, interferon regulatory factor 5 (IRF5), nuclear factor‐*κ*B (NF‐*κ*B), STAT3, STAT6, etc.), (3) regulating amounts and activity of micro‐RNAs (miR‐155 and miR‐125b, etc.), (4) inhibiting enzymes (histone deacetylases (HDACs), phosphoinositide 3‐kinase *γ* (PI3K*γ*), etc.).^[^ ^23^, ^34^, ^53^, ^81^, ^82^, ^83^ ^]^ A number of corresponding TAMs‐repolarization agents (including small molecule drugs, antibodies, agonists, or siRNAs, etc.) have been demonstrated to successfully promote the repolarization of M2‐like macrophages into M1‐like ones, however, their therapeutic efficacy is limited due to the poor tumor accumulation and off‐target adverse effects.^[^ ^23^, ^82^ ^]^ Therefore, nanotechnology has been harnessed for tumor‐targeting delivery of these agents due to the better tumor‐targeting performance.^[^ ^3^, ^5^, ^22^, ^23^ ^]^ Furthermore, some nanoparticles themselves own the intrinsic properties for repolarizing TAMs. The nanoparticles can repolarize TAMs through (1) their direct repolarization of TAMs via their intrinsic repolarization capability, and (2) their indirect mediated repolarization via modulating the TME and nanoparticles‐based immunotherapy.

#### 3.3.1 Delivering tumor‐associated macrophages reprogramming agents by nanoparticles

##### Regulating surface receptors of tumor‐associated macrophages

It has been well known that macrophages are highly plastic. They can maintain their different functional phenotypes according to their surrounding environment, and their functions are highly associated with receptor‐ligand‐mediated cell‐cell communications, in which the interactions between macrophage surface proteins with carbohydrate‐recognition domains (CRDs) and glycocalyx on the contacting cells are crucial.^[^ ^23^, ^84^ ^]^ Based on this mechanism, Su et al. developed the glycocalyx‐mimicking nanoparticles (glyco‐NPs) to interact with the surface factors CD301 or CD206 with CRDs via mimicking the specific receptor‐ligand interactions, and effectively repolarized M2‐like macrophages into M1‐like macrophages.^[^ ^84^ ^]^

TLRs, one class of pattern‐recognition receptors, can recognize highly conserved bacterial structures (such as, LPS) and bacteria‐ or viruses‐derived unmethylated CpG DNA, which can facilitate TAMs turning into M1 pro‐inflammatory phenotype.^[^ ^85^, ^86^, ^87^, ^88^, ^89^ ^]^ Therefore, multiple synthetic TLR agonists have been tested in preclinical models. For example, a TLR7/8 agonist 3M‐052 has been proved to potently reprogram M2‐like TAMs into anti‐tumor M1 phenotype, and result in regression of B16F10 melanoma.^[^ ^90^ ^]^ In addition, the TLR7 ligands (e.g., imiquimod and 852A) and TLR9 ligands (e.g., IMO‐2055) have been investigated in clinical trials.^[^ ^82^ ^]^ CSF‐1 and CSF‐1R not only play critical roles in monocyte recruitment and TAMs survival, but also induce the tumor‐supportive M2‐like TAMs, as demonstrated in a glioma model.^[^ ^91^ ^]^ In this study, the CSF‐1R inhibitor BLZ945 can repolarize TAMs by decreasing M2‐related markers, rather than deplete the macrophages, because glioma secreted granulocyte‐macrophage CSF (GM‐CSF) and IFN‐*γ* to support TAMs survival upon CSF‐1R inhibition.

**FIGURE 4:**
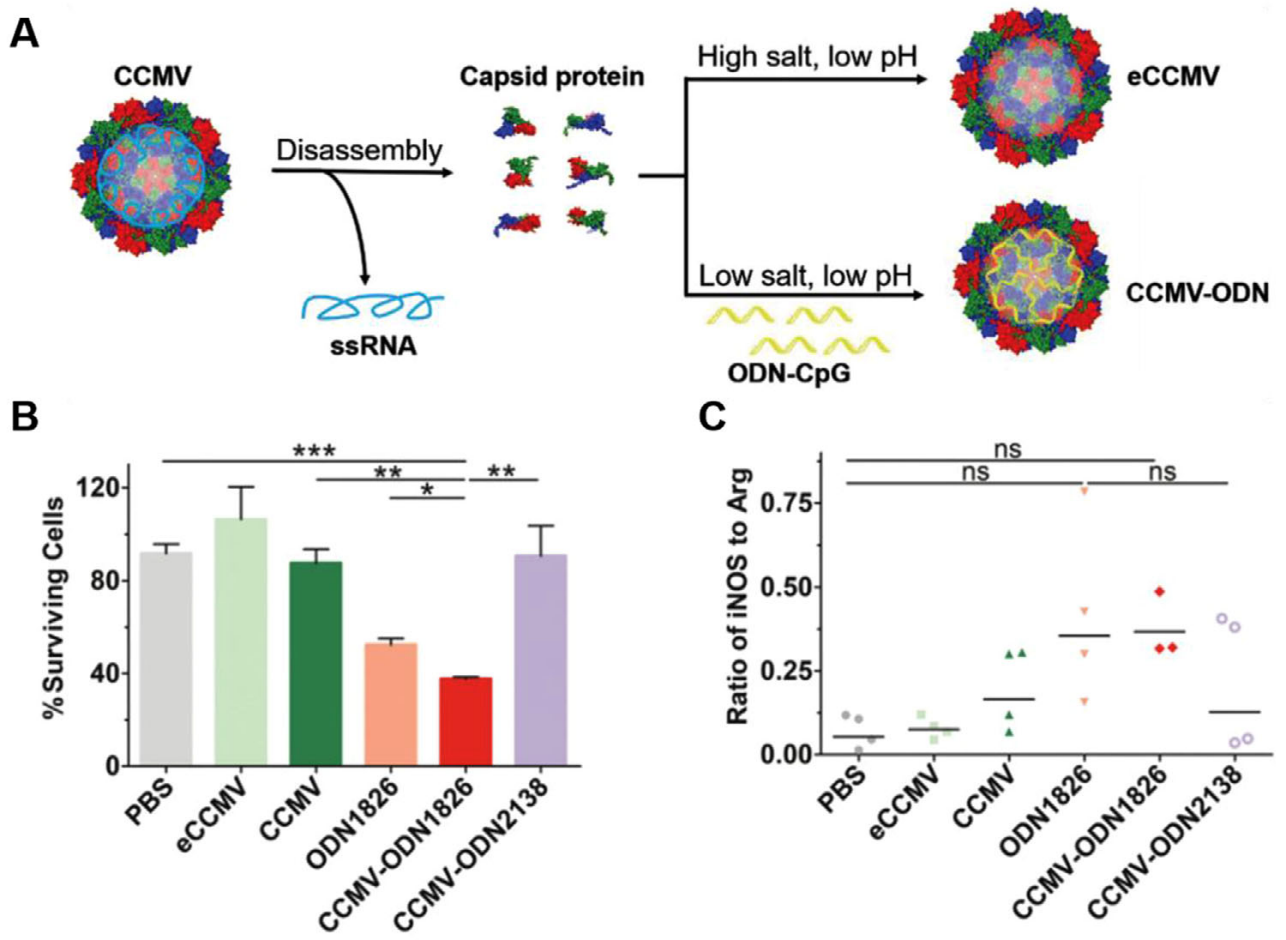
Fabrication of Cowpea chlorotic mottle virus (CCMV)‐based VLPs for delivery of ODN1826 to reprogram M2‐like TAMs. (A) Schematic illustration of CCMV disassembly and reassembly. (B) CCMV‐ODN1826 facilitated the death of CT26‐luc cells cocultured with TAMs. (C) The iNOS/Arg1 ratio in the TAMs of ODN1826‐treated CT26 tumors. Reproduced with permission.^[^ ^89^ ^]^ Copyright 2020, John Wiley & Sons

CpG is a motif of bacterial DNA or synthetic oligodeoxynucleotides (ODNs). CpG‐ODNs as a type of TLR9 agonists are always susceptible to degradation by nucleases in vivo, which can compromise the efficacy of CpG‐ODNs monotherapy and limit its application in TAMs‐repolarization.^[^ ^89^, ^92^, ^93^, ^94^ ^]^ In this context, virus‐like particles (VLPs) are good vector for nucleic acid delivery, because they are similar to the natural evolutionary viruses. Plant viruses have the merits of easy preparation and low cost, non‐infectiousness to mammals, and rapid internalization by immune cells especially APCs. Cai et al. designed cowpea chlorotic mottle virus‐based VLPs to deliver the type B CpG ODN1826 into TAMs (Figure 4).^[^ ^89^ ^]^ The resultant platforms enhanced phagocytosis of CpG ODN1826 in TAMs, and thus remarkably activated TLR9 to facilitate M2‐to‐M1 repolarization with enhanced iNOS/Arg ratio in the CT26 tumor‐bearing mice, which led to obvious tumor‐inhibiting efficacy and prolonged survival time of tumor‐bearing mice. Shan et al. devised a M2 macrophage‐targeting peptide (M2pep) for modification of human ferritin heavy chain (rHF) nanocages, which encapsulated the CpG ODNs for TAMs‐targeting administration.^[^ ^95^ ^]^ The resultant M2pep‐rHF‐CpG nanoparticles could significantly enhance tumor accumulation of CpG ODNs due to the TAMs‐targeting functions of both rHF and M2pep, and thus converted TAMs to anti‐tumor M1 phenotype, evidenced by an increased M1/M2 ratio in 4T1 tumors. In addition, M2pep‐rHF‐CpG nanoparticles were also suitable for the polarization of human macrophages, which was confirmed by the decreased M2‐associated IL‐10 maker and increased M1‐associated HLA‐DR, IL‐6, and TNF‐*α* markers in human macrophage THP‐1 cell line, and the elevated M1/M2 ratio in M2 type human peripheral blood mononuclear cells.

In addition to CpG, the TLR7/8‐agonists also have been widely explored. For example, the TLR7/8‐agonist R848‐loaded *β*‐cyclodextrin nanoparticles (CDNP‐R848) could be efficiently accumulated in tumor to re‐educate M2‐like TAMs, and inhibit the growth of MC38 tumor.^[^ ^96^ ^]^ Kim et al. designed a nanoemulsion (NE) based formulation loading with the TLR7/8 agonists (NE (TLR7/8a)), which could repolarize M2‐like TAMs into M1‐like ones and inhibit B16F10 tumor growth to prolong the survival of tumor‐bearing mice.^[^ ^97^ ^]^ Additionally, local administration of NE (TLR7/8a) with tumor antigens could also induce CD8^+^ T cell and NK1.1^+^ cell immunity and improve the efficacy of anti‐PD‐L1 therapy. Similarly, imidazoquinolines including imiquimod and resiquimod (R848), the ligands of both human TLR7 and TLR8 and mouse TLR7, have also been demonstrated to reprogram tumor‐promoting TAMs to a tumoricidal phenotype.^[^ ^98^, ^99^ ^]^ The anti‐MMR (mannose receptor) nanobody‐conjugated IMDQ (anti‐MMR Nb‐IMDQ) could efficiently target the MHC‐II^low^ MMR^high^ TAMs and reverse them to M1 pro‐inflammatory phenotype, resulting in great reduction of LLC‐OVA tumor progression.^[^ ^98^ ^]^ Similarly, the M2pep modified B16‐OVA tumor cell membrane covered poly‐(lactic‐*co*‐glycolic acid) (PLGA)‐encapsulating R848, designated as M2pep (PNP@R@M‐T), also could promote M2‐to‐M1 repolarization as well as infiltration and activation of CD8^+^ T cell population.^[^ ^99^ ^]^ In addition, the ginseng‐derived nanoparticles could activate both TLR4 and myeloid differentiation antigen 88 (MyD88) signaling to reset CD11b^+^F4/80^+^CD206^+^ M2‐like TAMs toward an anti‐tumor CD11b^+^F4/80^+^CD86^+^ M1 phenotype, and thereby eliciting CD8^+^ T cell immunity and the regression of mouse melanoma.^[^ ^100^ ^]^ Ramesh et al. designed a stable supramolecular structure named AK750, which sustainably blocked CSF‐1R to switch CD11b^+^CD206^+^M2‐like TAMs into CD11b^+^MHCII^+^CD80^+^CD86^+^ M1 phenotype in the B16F10 melanoma.^[^ ^101^ ^]^ Engineering anti‐SIRP*α* antibody with AK750 (anti‐SIRP*α*‐AK750) could further enhance TAMs‐targeting, repolarization and tumor‐phagocytosis capability. Similarly, the self‐assembled nanoparticles‐coloaded the inhibitors of CSF‐1R and Src homology region 2 (SH2) domain‐phosphatase SHP2 (DNTs) could also block both CSF‐1‐CSF‐1R and CD47‐SIRP*α* axes, which synergistically promoted M2‐to‐M1 repolarization and phagocytosis of tumors.^[^ ^102^ ^]^ Chen et al. developed tumor‐associated macrophage membrane (TAMM)‐coated photosensitizer‐conjugated upconversion nanoparticles (NPR@TAMM) to block CSF‐1‐CSF‐1R axis for TAMs‐repolarization.^[^ ^103^ ^]^ The resultant nanoparticles could specifically deplete CSF‐1 levels in the serum and distant tumor cells, because the primary tumor‐derived TAMs can directly bind with CSF‐1 by mimicking CSF‐1‐CSF‐1R interactions between macrophages and tumor cells. Then, combining with upconversion nanoparticles‐mediated PDT could successfully decrease M2‐related CD206, TGF*β*, Arg1, and IL‐10 markers, and increase M1‐related MHCII, IL‐12, and iNOS markers. More importantly, the CD4^+^ and CD8^+^ T cell immunity were also amplified to result in sufficient inhibition of 4T1 tumor progression.

##### Modulating transcription signals

Transcriptional regulation plays a vital role in maintaining phenotypes of macrophages, which are responsible for promoting their target genes’ transcription. The STATs, IRFs, NF‐*κ*B, activator protein 1 (AP‐1), CREB‐C/EBP axis and peroxisome proliferator‐activated receptor‐*γ* (PPAR*γ*) are the most popular transcription factors, in which STAT1, STAT5, IRF5, NF‐*κ*B, and AP‐1 are important for polarization of M1 pro‐inflammatory macrophages, while STAT3, STAT6, and IRF4, CREB‐C/EBP axis and PPAR*γ* are critical for promoting repolarization of M2 anti‐inflammatory phenotype.^[^ ^23^, ^53^, ^81^, ^104^ ^]^ STAT1 is a typical transcription factor involved in IFN‐*γ* induced M1‐polarization, and IFN‐*γ* induced STAT1 can elicit a potent anti‐tumor immunity in melanoma.^[^ ^105^ ^]^ Moreover, the immune checkpoint inhibitor T cell Ig mucin‐3 (Tim‐3) can maintain M2 phenotype of TAMs through inhibiting STAT1‐miR‐155 signaling pathway in a model of colon cancer.^[^ ^106^ ^]^ Creatine can also inhibit iNOS generation in macrophages by blocking IFN‐*γ*‐JAK‐STAT1 signaling.^[^ ^107^ ^]^ All these studies and results suggest the significant role of STAT1 in M1‐polarization.

Activating IRF5 is crucial for M1‐polarization and expression of pro‐inflammatory cytokines including IL‐12 and IL‐23.^[^ ^108^, ^109^ ^]^ In contrast, knocking down IRF5 would abolish these effects,^[^ ^108^ ^]^ which demonstrates that IRF5 is an important target for enhancing M1‐polarization. NF‐*κ*B is also a classical transcription factor for M1‐polarization as reported extensively.^[^ ^110^ ^]^ STAT6 is typically activated by IL‐4 and IL‐13 to induce M2‐like macrophages. Previous study reported that STAT6 inhibitor PM37 can prevent its activation by inhibiting its phosphorylation at tyrosine 641 (Y641), which resulted in an obvious decrease in M2‐related markers and prevention of M2‐mediated radioresistance in an inflammatory breast cancer model.^[^ ^111^ ^]^

**FIGURE 5:**
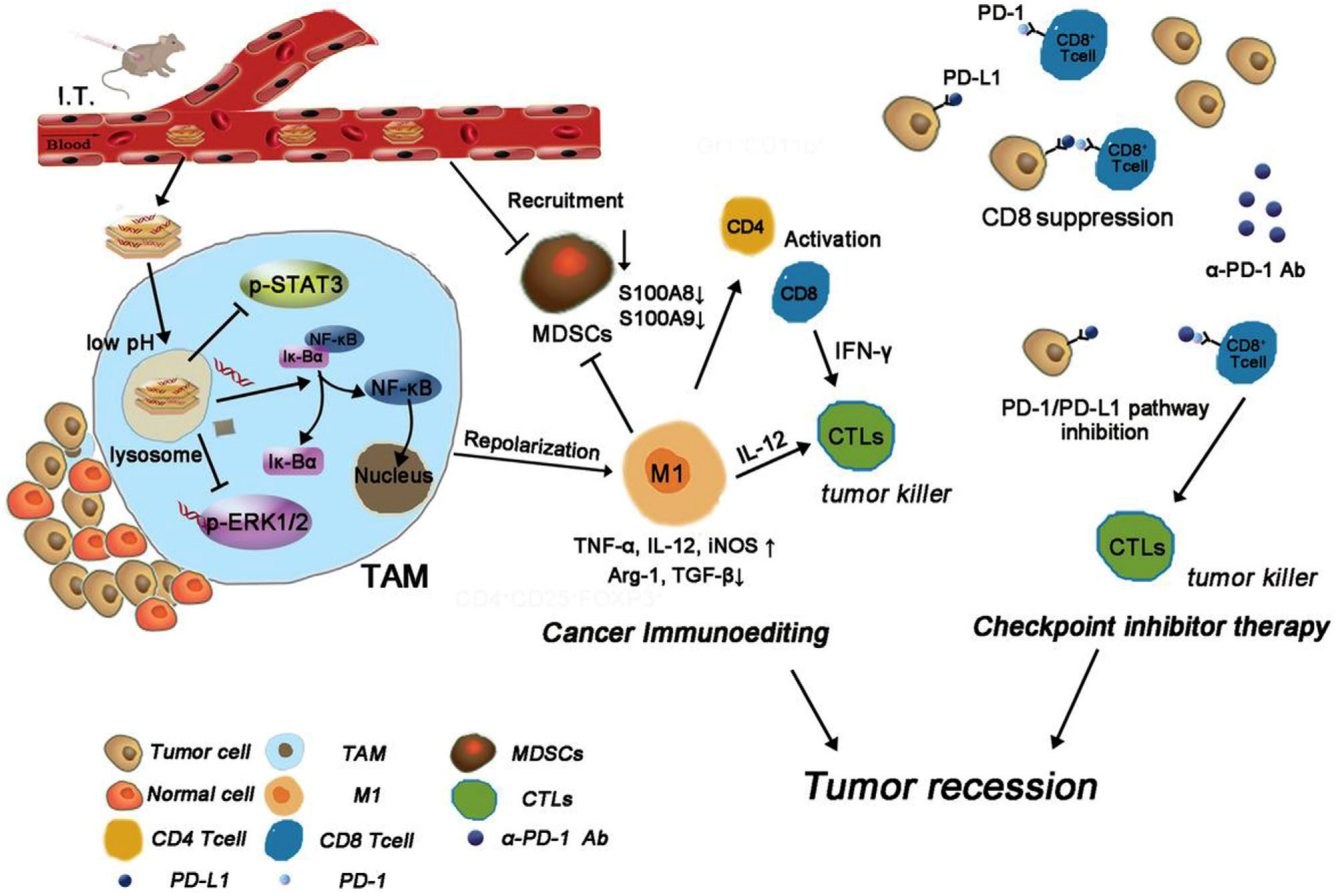
Schematic illustration of LDH@155 remodeling the immunoenvironment to enhance tumor immunotherapy. Reproduced with permission.^[^ ^113^ ^]^ Copyright 2019, John Wiley & Sons

Fu et al. developed a optogenetic system from conjugated polymer nanoparticles (CPNs) that encapsulated interferon heat‐shock‐promoter (HSP70) and IFN‐*γ* plasmid.^[^ ^112^ ^]^ Upon near‐infrared (NIR) light irradiation, the CPNs could act as a photothermal agent to activate HSP70 for facilitating the expression of downstream IFN‐*γ* gene in tumor cells to promote IFN‐*γ* secretion, which can selectively recognize and interact with IFN‐*γ* receptor (IFN‐*γ*R) expressed on surrounding M2‐like TAMs to repolarize them into tumor‐inhibiting M1 phenotype via IFN‐*γ*‐JAK‐STAT1 signaling pathway. Yang et al. utilized miR155‐loaded nanoscale layered double hydroxides (LDHs) (LDH@155) to treat TC‐1 tumor‐bearing mice (Figure 5).^[^ ^113^ ^]^ The results showed that LDH@155 induced repolarization of M2‐likeTAMs, and significantly weaken phospho‐STAT3 (p‐STAT3), phospho‐ERK1/2 (p‐ERK1/2) in TAMs in comparison with control group. In contrast, the M1‐related NF‐*κ*B proteins were obviously up‐regulated and NF‐*κ*B upstream inhibitor I*κ*‐B*α* was dramatically down‐regulated.^[^ ^114^ ^]^ In addition, NF‐*κ*B inhibitor (JSH‐23) could remarkably inhibit LDH@155‐mediated M2‐to‐M1 repolarization. All these results suggest that LDH@155 could synergistically suppress M2‐related signaling pathways and enhance M1‐related ones. In addition, LDH@155 could enhance anti‐PD‐1 immunotherapy and remarkably suppress TC‐1 tumor growth. Chen et al. developed dual‐targeting nanocarriers for delivering the STAT3 silencing siRNA to both tumor cells and TAMs.^[^ ^115^ ^]^ The gene expressions of STAT3 in tumor and TAMs cells were significantly down‐regulated, resulting in M2‐to‐M1 repolarization. In addition, the protein levels of STAT3 and p‐STAT3 in tumor tissues were significantly decreased and thus potently reduced M2‐like TAMs, as well as, LLC tumor progression. Xiao et al. designed a micellar nanodrug carrier for delivering I*κ*B kinase‐*β* (IKK*β*) siRNA and STAT6 inhibitor AS1517499 (AS), and modified with a M2pep to target M2‐like TAMs for treatment of 4T1 breast tumor.^[^ ^116^ ^]^ The resultant ST‐AS&Si nanodrug down‐regulated the expressions of IKK*β* (a critical upstream factor controlling NF‐*κ*B activation) and phospho‐STAT6 (p‐STAT6) in M2‐like macrophages, thereby repolarizing them into tumor‐killing M1 phenotype. The ST‐AS&Si treatment robustly amplified anti‐tumor T cell immunity via increasing tumor‐infiltrating CD8^+^ T cells and decreasing immunosuppressive Treg cells, leading to remarkable suppression of tumor progression and metastasis.

To induce NF‐*κ*B‐mediated M1‐polarization, Zhao et al. found that the nanocomposites of ferumoxytol (FMT) and TLR3 agonist poly (I : C) (PIC) (denoted as FP‐NPs) could attenuate melanoma progression and lung metastasis by promoting NF‐*κ*B signaling‐mediated M1‐polarization, which decreased M2 related CD206, Arg1 markers and increased M1‐related CD86, iNOS, TNF‐*α* markers, as well as, NO generation.^[^ ^117^ ^]^ In addition, the FP‐NPs could augment tumor‐phagocytosis of macrophages via NOX2‐derived ROS production. Chen et al. fabricated a photothermal‐activatable in situ self‐assembled nanomicelle dissolving microneedle (DMN) patch based on hyaluronic acid (HA) to deliver autophagy inhibitor (chloroquine, CQ) and ICD‐inducer (IR780) to deep tumor via the interaction of HA with CD44 receptor.^[^ ^118^ ^]^ The resultant nanomicelle could promote M2‐like TAMs‐repolarization through CQ‐mediated autophagy inhibition to activate NF‐*κ*B signaling pathway and thus suppress the primary and distant melanoma tumor progression in synergy with localized photoimmunotherapy.

Combining the suppression of transcription factors that induce M2 phenotype with the activation of transcription factors that promote M1‐polarization is also an attractive strategy. Zhang et al. developed glycocalyx‐mimicking nanoparticles (GNPs), containing the amphiphilic deblock copolymers poly (mannopyranoside/galactopyranoside methacrylate)‐block‐polystyrene, to modulate the phenotype of TAMs.^[^ ^119^ ^]^ The resultant GNPs can enhance the surface carbohydrates density, which promoted specific targeting of TAMs via binding to lectin receptors through the “cluster glycoside effect,” and then polarize them into anti‐tumor M1 phenotype via suppressing phosphorylation of STAT6 and activating phosphorylation of NF‐*κ*B. This synergistic strategy also benefited to the improvement of anti‐PD‐L1 immunotherapy, and induction of remarkable enhanced tumor‐infiltrating CD4^+^ and CD8^+^ T cells, which resulted in dramatical regression of LLC tumor.

Since IRF5 is a potent M1‐inducer, Liu et al. developed M1‐like macrophage membranes‐coated PLGA‐encapsulating Fe_3_O_4_ NPs and TLR7 agonist imiquimod (R837) (PLGA‐ION‐R837@M (PIR@M)), and found that the resultant nanocarrier could efficiently target TAMs to facilitate M2‐like TAMs‐repolarization‐mediated anti‐tumor therapeutic effect and CD8^+^ T cells’ infiltration in mouse breast tumor. Furthermore, the M2‐to‐M1 repolarization function of PIR@M nanocarrier was attribute to both Fe_3_O_4_ NPs‐mediated IRF5 singling pathway and R837‐initiated NF‐*κ*B signaling pathway.^[^ ^120^ ^]^ In recent years, in vitro‐transcribed mRNA, which can deliver genetic information directly into target cells and induce transient expression of specific proteins, have attracted much attention.^[^ ^121^, ^122^ ^]^ Zhang et al. fabricated mRNA nanocarriers from cationic poly(*β*‐amino ester) (PbAE) polymers, IRF5 mRNA and IKK*β* mRNA (kinase for promoting IRF5 phosphorylation and activation), and then engineered them with a Di‐mannose moiety.^[^ ^122^ ^]^ The resultant mRNA‐PbAE complex could successfully promote gene and protein expressions of IRF5 in macrophages from the ovarian and melanoma tumor models. In the ovarian tumor, the mRNA‐PbAE complex could significantly reduce Ly6C^–^F4/80^+^CD206^+^ M2‐like macrophage population (2.6% ± SE/2.1% vs. 43% ± SE/15.6% in controls) and enhance M1‐like macrophage population (from 0.5% ± SE/0.2% to 10.2% ± SE/4.1%), then increase pro‐inflammatory IL‐12 (3.4‐fold higher), IFN‐*γ* (8.4‐fold higher), and TNF‐*α* (1.5‐fold higher) cytokines and tumor infiltration of T cells (i.e., CD8^+^ T cells, 10.6‐fold and CD4^+^ T cells, 3,5‐fold). It could also transfer CD206^+^MHCII^−^CD11c^+^CD11b^low^) macrophages into activated CD206^−^ MHCII^+^CD11c^−^CD11b^+^ phagocytes in melanoma. These studies suggest that targeting TAMs‐regulator‐encoding genes can be a good choice for repolarizing tumor‐supportive macrophages into anti‐tumor phenotype.

##### Regulating amounts and activity of microRNAs

MicroRNAs (miRNAs) are small, endogenous non‐coding RNAs with a length of 20 to 24 nucleotides.^[^ ^123^, ^124^ ^]^ They can regulate gene expression at post‐transcriptional process and participate in cell proliferation, metabolism apoptosis, and differentiation.^[^ ^124^, ^125^ ^]^ Correspondingly, miRNAs have been confirmed to control macrophage polarization and different phenotypes of macrophage with distinct characteristic miRNA profiles,^[^ ^123^, ^124^, ^126^ ^]^ in which miR‐155, miR‐125b, miR‐21, miR‐127, and miR‐9 can promote M1‐polarization, miR‐146a, miR‐34a, miR‐223, miR‐124, miR‐132, miR‐125a‐5p, and let‐7c can facilitate M2‐polarization.^[^ ^123^, ^124^, ^127^ ^]^ More importantly, the RNase‐III enzyme DICER is a crucial regulator for the maturation of miRNAs.^[^ ^128^ ^]^ Therefore, up‐regulating M1‐related miRNAs or down‐regulating M2‐related miRNAs and targeting DICER can be good choices for modulating macrophage phenotypes.

The miR‐155 can effectively reprogram tumor‐supportive M2‐like TAMs to anti‐tumor M1 phenotype by up‐regulating expressions of miR‐155 in TAMs, M1‐related IL‐12, iNOS, and MHC II markers, and suppressing M2‐related Msr2 and Arg1 markers, which resulted in an obvious regression of B16 tumor.^[^ ^129^ ^]^ Huang et al. demonstrated that cypermethrin (CYM) can facilitate M2‐like macrophages polarization by inhibiting expression of miR‐155, which subsequently triggered Lewis lung cancer cells metastasis.^[^ ^130^ ^]^ It has been demonstrated that enforcing expression of miR‐125b can make macrophage susceptible to IFN‐*γ* stimulation by inhibiting expression of IRF4, which is a positive modulator of M2‐like macrophages.^[^ ^53^, ^131^, ^132^ ^]^ Additionally, depletion of DICER, a miRNA‐processing enzyme, can promote repolarization of M2‐like TAMs into M1 phenotype in MC38 and LLC tumor models.^[^ ^133^ ^]^

Zang et al. designed a lipid‐coated calcium phosphonate nanoparticles‐based miRNA delivery system (CaP/miR@pMNPs), which was modified with mannose for TAMs‐targeting delivery of miR155.^[^ ^134^ ^]^ The resultant CaP/miR@pMNPs decreased M2‐associated IL‐10, Arg1, MMP9, and vascular endothelial growth factor (VEGF) makers and increased M1‐associated IL‐12 and iNOS makers, both which resulted in great tumor suppression and prolonged survival times of tumor‐bearing mice. Parayath et al. developed hyaluronic acid‐poly(ethylenimine) (HA‐PEI)‐based nanoparticles, which encapsulated miR‐125b for targeting CD44^+^ macrophages.^[^ ^135^ ^]^ The HA‐PEI‐125b nano‐formulation could effectively accumulate in peritoneal macrophages after intraperitoneal (i.p.) administration, which could deliver HA‐PEI‐125b nanoparticles to the lungs due to the targeting capability of peritoneal macrophages to inflammatory tumor tissue and thereby successfully reprogramed M2‐like lung TAMs into M1‐like ones. Correspondingly, the iNOS (M1‐rekated marker)/Arg1 (M2‐related marker) ratio was increased by 300 folds and the M1/M2 ratio was increased more than 6 folds. Talekar et al. fabricated HA‐based nanoparticles (NPs) for co‐delivering wild‐type (wt‐) p53 and microRNA‐125b (miR‐125b) plasmid DNA to transfect SK‐LU‐1 human lung adenocarcinoma.^[^ ^136^ ^]^ This strategy significantly up‐regulated wt‐p53 and miR‐125b gene expression and thus promoted cell apoptosis. Finally, the iNOS‐Arg1 ratio (M1/M2) in J774.A1 murine macrophages was obviously increased after co‐culturing with above transfected SK‐LU‐1 cells. Ultimately, the wt‐p53‐mediated tumor cell apoptosis and miR‐125b‐mediated M2‐repolarization successfully regressed tumor progression. Similarly, the exosomes isolated from M1‐polarizing miRNAs transfected tumor cells also can promote M1‐polarizaion.^[^ ^132^, ^137^ ^]^ Trivedi et al. developed dual‐targeted hyaluronic acid‐based nanoparticles‐encapsulating wt‐p53 and miR‐125b to transfect the KRAS/p53 mutant SK‐LU‐1 non‐small cell lung cancer (NSCLC) cells.^[^ ^137^ ^]^ They found that the SK‐LU‐1‐isloated exosomes also could transfer TAMs into M1 profiles.

In addition to single miRNA, human pancreatic cancer (Panc‐1) cell‐derived exosomes, which were co‐transfected by HA‐PEI/HA‐PEG nanoparticles‐transmissive miR‐155 and miR‐125b2 plasmid DNA, also played synergistic roles in reprogramming the M2‐like macrophages into M1 phenotype.^[^ ^132^ ^]^ It should be noted that nanovesicles (NVs) can be a good substitute for exosomes because they can be fabricated with a higher yield, richer protein and RNAs than exosomes. Choo et al. developed a type of M1‐like macrophages‐derived exosome‐mimetic nanovesicles (M1NVs), which could target tumor due to the enhanced expression of leukocyte‐derived adhesion molecules (e.g., lymphocyte function‐associated antigen 1, LFA‐1) on M1NVs, and efficiently polarize M2‐like TAMs into M1 phenotype.^[^ ^127^ ^]^ The M1NVs increased M1‐related makers (including CD86, IL‐6, TNF‐*α*, and iNOS), up‐regulated M1‐related miRNAs (including miR‐155, miR‐125, and miR‐21) and down‐regulated M2‐related miRNAs (miR‐34a, let‐7c, and let‐7f) compared to the unstimulated M0NVs. Therefore, they could potently promote M2‐to‐M1 repolarization and amplify the anti‐tumor capability of anti‐PD‐L1 antibody (a‐PD‐L1).

##### Inhibiting enzymes

HDACs are epigenetic regulators, which can trigger histone and nonhistone proteins de‐acetylation and thus modulate gene expression.^[^ ^138^, ^139^ ^]^ Generally, decreased acetylation can strengthen the binding of DNA to histones and trigger transcriptional repression, while increased acetylation can promote transcription factor activity for transcriptional induction.^[^ ^139^, ^140^ ^]^ The gene expression profiles (i.e., pro‐ and anti‐inflammatory genes) in LPS or IFN‐*γ* stimulated macrophages usually depend on transcriptional, post‐transcriptional, and epigenetic modulation. Therefore, histone modification is a key type epigenetic regulation involving in regulating macrophage activation and polarization.^[^ ^140^ ^]^ It has been found that inhibition of HDAC can effectively promote reprogramming of TAMs.^[^ ^141^, ^142^, ^143^, ^144^ ^]^ TMZ195, a well‐known inhibitor of Class IIa HDACs, can modulate tumor‐supportive TAMs into anti‐tumor phenotype with enhanced expression of CD40 and phagocytic capability in the luminal B‐type breast tumor model.^[^ ^141^ ^]^ Additionally, the low‐dose HDAC inhibitor (HDACi), trichostatin‐A (TSA) could also polarize tumor‐supportive TAMs into tumoricidal M1 phenotype by decreasing M2‐related Arg1, CD206, and Fizz1 markers and increasing M1‐related Nos2 and IL‐6 markers.^[^ ^142^ ^]^ More importantly, the TSA can block recruitment of immunosuppressive MDSCs, and display enhanced synergistic anti‐tumor effect by combining with anti‐PD‐L1 immunotherapy. Since vorinostat (Vor) is a popular HDACi approved by FDA in 2006,^[^ ^145^ ^]^ Peng et al. developed a trastuzumab‐modified and mannosylated liposomal delivery system for co‐delivering Vor and Gefitinib (Gef), and named it as tLGV, which could target both M2‐like TAMs and HER2‐positive NSCLC cells. The down‐regulation of HDAC2 decreased M2‐like macrophage population.^[^ ^143^ ^]^ Accordingly, M1‐like macrophages were elevated, which benefited to the increase of ROS level in tumors and subsequent regulation of the intracellular redox balance through ROS/NOX3/MsrA axis, leading to resensitization of the EGFR^T790M^‐positive NSCLC to gefitinib and amplified anti‐tumor effects. Similarly, since simvastatin (Siv) is a cholesterol‐lowering agent which can reprogram TAMs by regulating cholesterol metabolism,^[^ ^146^ ^]^ Tu et al. developed a deformable liposome system (D‐Lipo) to co‐deliver Vor and Siv for treating the NSCLC.^[^ ^144^ ^]^ In vitro, Vor treatment alone could obviously decrease M2‐related CD206 maker via inhibiting HDAC2 expression and STAT6 phosphorylation. Siv treatment alone could also reprogram M2‐like macrophages by blocking the expression of LXR‐*α*/ABCA1 involving in cholesterol metabolism‐mediated macrophage polarization. In vivo, the co‐delivery system could synergistically enhance repolarizing effect of M2‐like TAMs, which was better than that of individual Vor or Siv treatment. Overall, this treatment could benefit to activation of cytotoxic Granzyme B^+^, Ki67^+^, or IFN‐*γ* ^+^ CD8^+^ T cells, inhibition of immunosuppressive Treg cells and angiogenesis.

PI3K*γ*, a gamma isoform of PI3K, plays a critical role in controlling immune suppression.^[^ ^147^ ^]^ It has been demonstrated to be an important immune checkpoint for mediating M2‐polarization in many types of tumors such as gastric cancer, PDAC, breast carcinoma, and B16‐GMCSF melanoma.^[^ ^148^, ^149^, ^150^ ^]^ It has been found that PI3K*γ* could up‐regulate anti‐inflammatory factors expression (i.e., TGF‐*β*, IL10, and Arg1), while down‐regulate pro‐inflammatory factors (i.e., IL‐12 and TNF‐*α*) expression through inducing AKT, mTOR, and C/EBP*β* activation, and inhibiting NF‐*κ*B activation.^[^ ^147^, ^148^ ^]^ Jian‐pi‐yang‐zheng Decoction (JPYZ) is a classical Chinese medicine for treatment of advanced gastric cancer. Yuan et al. modified JPYZ to inhibit the activity of PI3K*γ*, which decreased IL‐10, increased TNF‐*α* and IL‐1*β* and ultimately reversed the M2‐like TAMs to anti‐tumor M1 phenotype in the gastric cancer through inhibiting the expression of p‐AKT, p‐I*κ*K*α*/*β*, p‐C/EBP*β*, and up‐regulating the expression of p‐NF‐*κ*B.^[^ ^148^ ^]^ Kaneda et al. found that blocking PI3K*γ* with either genetic (p110*γ*‐/‐) or pharmacological (TG100‐115, a PI3K*γ* inhibitor) promoted the repolarization of TAMs into M1‐like macrophages and thus activated tumor‐specific CD8^+^ T cells, which could effectively inhibit PDAC progression, invasion, metastasis, and desmoplasia.^[^ ^149^ ^]^ Henau et al. also demonstrated that blocking PI3K*γ* down‐regulated immunosuppressive M2‐associated TGF‐*β*, Arg1, and IDO markers, and up‐regulated immuno‐activated M1‐associated IL‐12 and iNOS markers in IPI‐549 treated 4T1 and B16‐GMCSF tumors.^[^ ^150^ ^]^ Collectively, these findings suggest that PI3K*γ* plays a crucial role in inducing immunosuppressive TAMs.

Li et al. designed a nanoplatform delivery system from porous hollow iron oxide nanoparticles (PHNPs) and carbonylated mannose for targeting delivery of PI3K*γ* small molecule inhibitor (3‐methyladenine, 3‐MA).^[^ ^151^ ^]^ The resultant nanoparticles (PHNPs@DPA‐S‐S‐BSA‐MA@3‐MA) could effectively target M2‐like TAMs and reverse them into anti‐tumor M1 phenotype by inhibiting PI3K*γ* expression, accompanied with up‐regulation of NF‐*κ*B p65 protein. As a result, the immunosuppressive TIME was normalized, which was evidenced by enhanced immune‐promoting immune cells (i.e., CD8^+^ T cells, CD4^+^ T cells, B cells NK cells) and factors (i.e., iNOS, IL‐1*β*, and TNF‐*α*), as well as, decreased immunosuppressive cells (i.e., Treg cells) and factors (i.e., IL‐10, TGF‐*β*, and Arg I). The normalization of immunosuppressive TIME resulted in successful regression of MDA‐MB‐231 tumor. In addition, enhanced immunotherapy can be achieved by combining blocking PI3K*γ* checkpoint with other TAMs‐targeting therapy. As mentioned previously, blocking CSF‐1R‐CSF‐1 axis is also an effective TAMs‐repolarizing method,^[^ ^152^ ^]^ Li et al. reprogramed tumor‐supportive M2‐like TAMs by combining PI3K*γ* inhibition with CSF‐1R inhibition.^[^ ^153^ ^]^ They designed TAMs‐targeting peptide (M2pep) modified nanomicell to encapsulate small molecule PI3K*γ* inhibitor BEZ235 and CSF‐1R siRNA. The nanomicelle could be significantly endocytosed by M2‐like TAMs to polarize them into M1‐like ones via synergistically blocking PI3K*γ* and decreasing CSF‐1R expression. Moreover, PI3K*γ* blockade could also inhibit immunosuppressive tumor‐infiltrating MDSCs. The M2‐like TAMs‐repolarizing system significantly induced potent anti‐pancreatic tumor immunity and effects.

#### 3.3.2 Tumor‐associated macrophages‐repolarization by intrinsic capability of nanoparticles

##### Direct repolarization by nanoparticles

**FIGURE 6:**
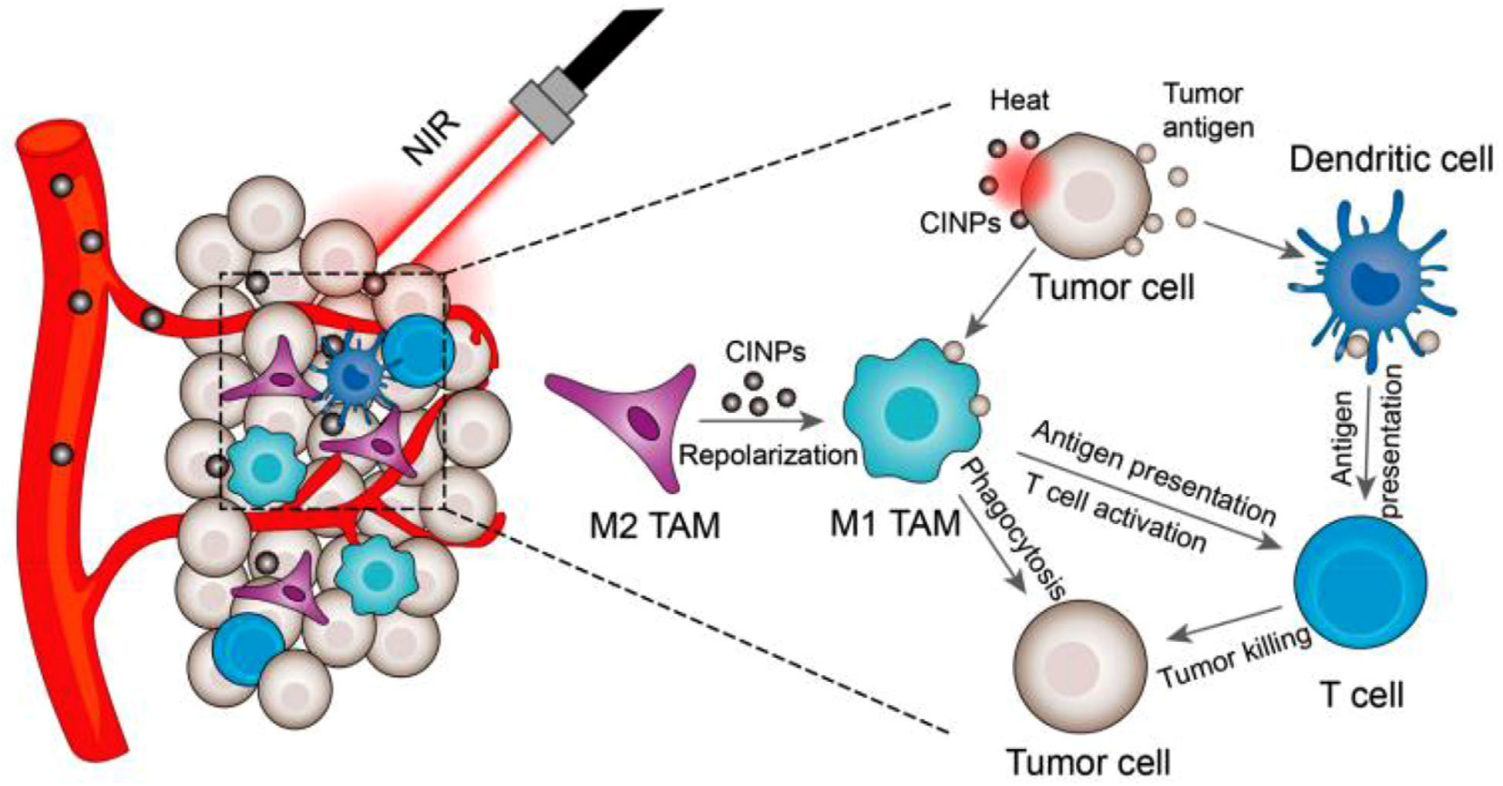
Schematic illustration of CINPs‐mediated M2‐like TAMs‐repolarization synergizing with photothermal therapy to suppress tumor growth. Reproduced with permission.^[^ ^158^ ^]^ Copyright 2019, American Chemical Society

Although most studies have focused on nanoparticles as delivery vehicles for TAMs‐reprograming agents, the intrinsic capability of nanoparticles on TAMs‐repolarization has been investigated recently. Zanganeh et al. found that the Food and Drug Administration (FDA)‐approved iron supplement ferumoxytol could intrinsically facilitate M2‐to‐M1 repolarization.^[^ ^154^ ^]^ Since M1‐like macrophages can release hydrogen peroxides for iron‐Fenton reaction to generate hydroxyl radical (OH·), the hydrogen peroxide increased 11‐fold and OH· increased 16‐fold in stimulated tumor cells. Co‐cultured macrophages with ferumoxytol increased apoptosis of tumor cells. The in vivo results showed that ferumoxytol‐mediated M1‐polarization could successfully prevent early mammary tumor progression and metastasis. Moreover, Gu et al. prepared magnetite IONPs (Fe_3_O_4_@D‐SiO_2_, iron (II, III)) and hematite IONPs (Fe_2_O_3_@D‐SiO_2_, iron (III)) to explore the mechanism of iron oxide‐mediated M1‐polarization. They found that magnetite IONPs, rather than hematite IONPs, could effectively induce M2‐to‐M1 repolarization, which specifically depended on IRF5 signaling pathway mediated by TNF receptor‐associated factor 6 (TRAF6)‐ubiquitination to achieve effective inhibition of melanoma tumor growth.^[^ ^155^ ^]^ Similarly, Jiang et al. fabricated platelet membrane‐camouflaged magnetic nanoparticles (Fe_3_O_4_‐SAS@PLT), which encapsulated sulfasalazine (SAS) in the mesoporous magnetic nanoparticles (Fe_3_O_4_).^[^ ^156^ ^]^ The resultant Fe_3_O_4_‐SAS@PLT nanoparticles could elicit ferroptosis‐mediated M2‐to‐M1 repolarization, which improved PD‐1 blockade immunotherapy and efficiently inhibited 4T1 metastatic tumors progression. Li et al. built hyaluronic acid‐decorated superparamagnetic iron oxide nanoparticles (HIONs)‐stimulated artificially reprogramming macrophages (HION@Macs), which could maintain activation of M1 macrophages due to iron ions‐induced activation of NF‐*κ*B.^[^ ^157^ ^]^ M1‐like macrophages can not only generate ROS, TNF‐*α*, and NO, for triggering tumor cell apoptosis and serve as signaling modulators to induce immune activation, but also can resist intratumoral M2‐inducing regulators and maintain activated state. Therefore, the HION@Macs could significantly increase M1‐related CD86, TNF‐*α*, and iNOS markers and decrease M2‐related CD206 marker, as well as, potent dendritic cells (DCs) maturation and tumor infiltration of CD8^+^ T cells. Moreover, all these activated anti‐tumor immune responses were further amplified upon magnet guidance, leading to dramatical regression of 4T1 breast tumor. Deng et al. extracted natural nanoparticles from cuttlefish ink (CINPs), which could reverse M2‐like TAMs to anti‐tumor M1 populations via activating mitogen‐activated protein kinase signaling pathway (Figure 6).^[^ ^158^ ^]^ Under the NIR irradiation, CINPs could exert excellent photothermal effect and amplified TAMs‐repolarization effects and activation of tumor‐specific CD8^+^T cells to elicit excellent anti‐tumor responses.

It has been found that low molecular weight HA could induce classical M1‐like macrophages.^[^ ^159^ ^]^ Zhang et al. proved that low molecular weight HA modified black phosphorus (BP) nanoparticles (HA‐BP) could successfully reeducate M2‐like macrophages into M1 phenotype with down‐regulated CD206 and increased CD86 expression, which was closely related to the capability of HA facilitating M1‐polarization.^[^ ^160^ ^]^ The HA‐BP could markedly reprogram tumor‐supportive TAMs and reduce 4T1 tumor growth. More importantly, the responses were further amplified by combining the BP‐mediated PTT and PDT upon 808 and 635 nm laser irradiation, suggesting that HA can be used to endow nanoparticles with functions of TAMs‐repolarization.

##### Indirect repolarization by nanoparticles

Modulating tumor microenvironment to reprogram tumor‐associated macrophages. The phenotype and function of macrophages are highly plastic and susceptible to the TME. They can transfer into tumor‐supportive M2 phenotype in response to the abnormal cues such acidity and hypoxia in the TME, or turn into anti‐tumor M1 phenotype by ROS.^[^ ^23^, ^161^, ^162^, ^163^ ^]^ Therefore, developing nanotechnology to modulate the TME holds great promise for TAMs‐repolarization‐mediated tumor immunotherapy.

Relieving acidic tumor microenvironment. Aerobic glycolysis is the main metabolic pathway of malignant tumors. It can heavily consume glucose to produce more acidic products such as hydrogen ions (H^+^) and lactic acid, which can be sensed by TAMs to facilitate an tumor‐supportive phenotype.^[^ ^52^, ^164^, ^165^, ^166^ ^]^ Therefore, relieving acidic TME via eliminating the source of lactic acid/ H^+^ (i.e., targeted suppression of aerobic glycolysis pathway to block intracellular lactic acid generation), or the acidic products (i.e., scavenge H^+^, inhibition of intracellular lactic acid/H^+^ efflux or exhaustion of extracellular existed lactic acid) can be a good option to reverse TAMs into M1 phenotype. Chen et al. prepared a type of fibrinogen solution consisting of anti‐CD47 antibody, calcium carbonate nanoparticles (aCD47@CaCO_3_) and thrombin solution. The fibrinogen solution could be sprayed at tumor surgical site to immediately form fibrin gel.^[^ ^165^ ^]^ The aCD47@CaCO_3_@fibrin could response to acid and sufficiently scavenge H^+^ at the surgical site of B16F10 tumor to allow M2‐to‐M1 repolarization. Meanwhile, the released anti‐CD47 antibody could elicit enhanced tumor‐phagocytosis of macrophages via blocking the CD47‐SIRPα checkpoint. Moreover, DCs were endowed with a higher tumor‐specific antigen presentation ability, and the aCD47@CaCO3@fibrin induced a stronger tumor‐specific T cell‐mediated suppression ability, and a lower metastasis.

The components involving in production of lactic acid/H^+^ in the TME include glucose transporters (i.e., GLUT1), glycolysis‐related enzymes (i.e., hexokinase II (HK2), pyruvate kinase M2 (PKM2), lactate dehydrogenase A (LDHA), lactate transporters (i.e., monocarboxylate transporters, MCT) and pH modulators (i.e., carbonic anhydrase, CAIX).^[^ ^167^ ^]^ On this basis, Wang et al. utilized shikonin (SHK) and JQ1‐co‐delivering mannosylated lactoferrin nanocarrier (Man‐LF NPs) to treat CT26 tumor.^[^ ^168^ ^]^ Due to the potential of SHK in inhibiting PKM2 to decrease the generation of lactate, and JQ1‐mediated inhibition of PD‐L1 expression, the Man‐LF NPs remarkably increased TAM1/TAM2 ratio, and potentiated tumor‐specific T cell immunity, which led to great reduction of CT26 tumor and prolonged survival time of tumor‐bearing mice. By taking the merit of MCT‐4 transporting lactate out of tumor cells for maintaining extracellular acidic TME,^[^ ^169^, ^170^ ^]^ Li et al. fabricated a cascade‐responsive nanoplatform via loading hydroxycamptothecin (HCPT) and siMCT‐4 into GSH‐responsive hollow mesoporous organosilica (HMONs@HCPT‐BSA‐PEI‐CDM‐PEG@siMCT‐4) to potentiate chemo‐immunotherapy.^[^ ^170^ ^]^ The results showed that siMCT‐4 mediated silence of MCT‐4 expression in B16F10 and 4T1 tumors, resulting in increased accumulation of lactate in tumor cells and decreased extracellular lactate level in TME. Therefore, this nanoplatform dramatically reversed CD206^+^F4/80^+^CD11b^+^ TAMs to CD86^+^F4/80^+^CD11b^+^ M1 phenotype, decreased immunosuppressive FOXP3^+^CD4^+^CD25^+^ Treg cells, and increased anti‐tumor IFN‐*γ* ^+^CD8^+^ T cells in B16F10 and 4T1 tumor‐bearing mice. In addition, the increasing lactate in tumor tells would induce tumor cell acidosis and apoptosis.^[^ ^169^, ^170^, ^171^ ^]^ Therefore, MCT‐4‐silence‐mediated tumor accumulation of lactate combining with HCPT successfully promoted tumor cell apoptosis to achieve great regression of primary and metastatic tumor.

**FIGURE 7:**
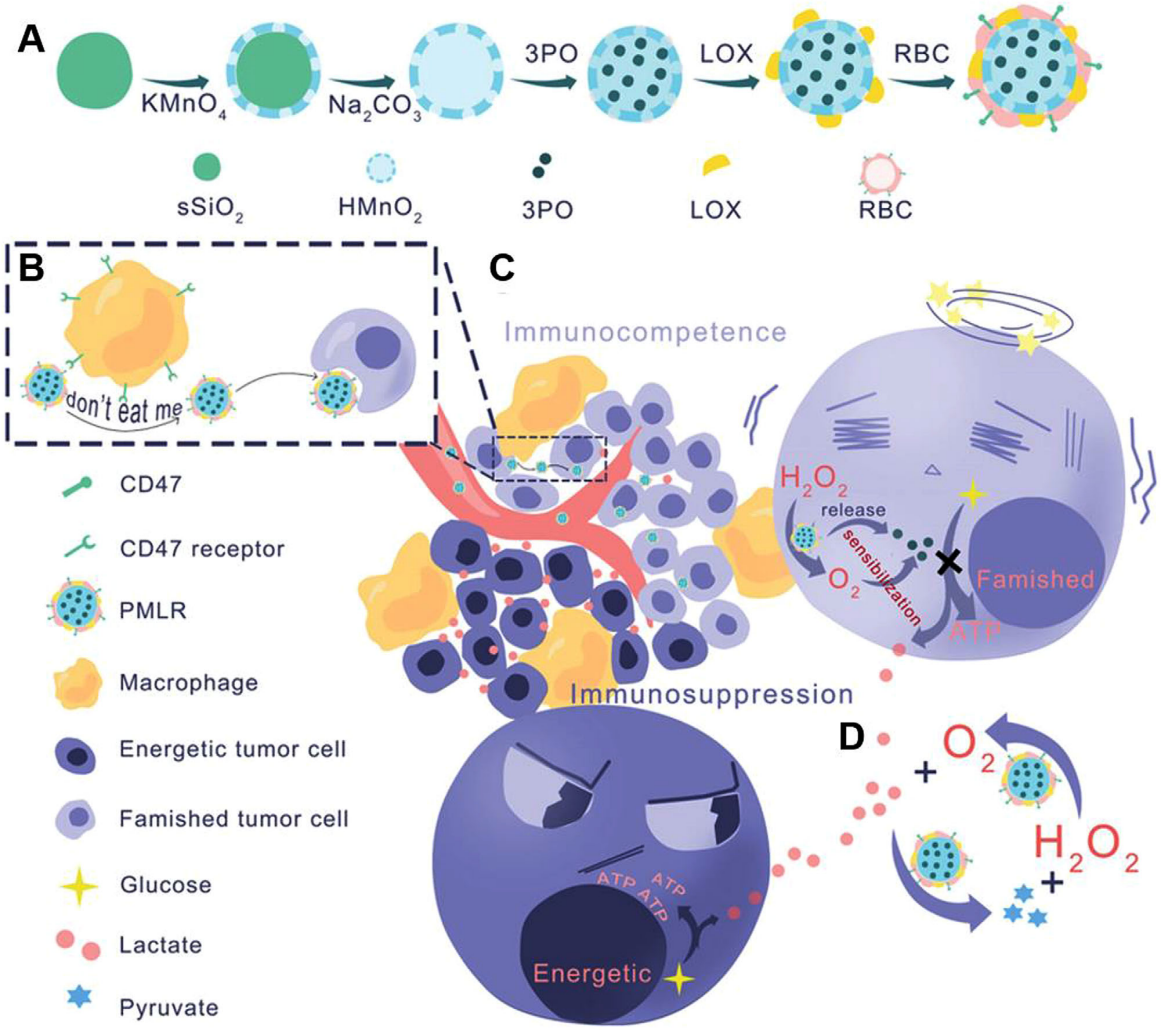
Schematic illustration of engineering PMLR nanoplatform to exhaust intra/extracellular lactic acid for alleviating immunosuppressive TME. (A) Synthesis of PMLR nanoplatform. (B) The inhibition of macrophage‐mediated engulfment by mRBC‐camouflaged nanoplatform mimicking the CD47‐mediated “don't eat me” signal. (C) The release of 3PO by PLMR system to block glycolysis of tumor. (D) The Cascade catalytic reaction of PLMR nanoplatform. PLMR initiated LOX‐mediated oxidation of lactic acid by O_2_ to generate H_2_O_2_ and pyruvate, in which H_2_O_2_ could react with HMnO_2_ to sustainably generate O_2_ for lactic acid oxidation. Reproduced with permission.^[^ ^172^ ^]^ Copyright 2019, John Wiley & Sons

In another strategy, the cascade catalytic (PMLR) nanosystem, which was prepared by coating lactate oxidase (LOX) and 3‐(3‐pyridinyl)‐1‐(4‐pyridinyl)‐2‐propen‐1‐one (3PO, a glycolysis inhibitor)‐loaded into hollow MnO_2_ (HMnO_2_) nanoparticles with red blood cell membrane (mRBC), could synchronously exhaust intra/extracellular lactic acid through 3PO mediated blockade of lactic acid source and LOX‐triggered extracellular lactic acid exhaustion (Figure 7).^[^ ^172^ ^]^ In addition, the mRBC can avoid phagocytosis of PMLR nanosystem by macrophages via mimicking the “don't eat me” checkpoint. LOX was used to catalyze the lactic acid oxidation with O_2_, and their by‐product could be catalyzed by HMnO_2_ to constantly supply O_2_ for lactic acid oxidation, resulting in an effective cascade catalytic reaction. Therefore, the dramatical decrease of lactic acid benefited to M2‐to‐M1 repolarization, successfully decreased F4/80^+^CD206^+^ M2‐like TAMs from 38.4% to 21.2% and increased M1/M2 ratio. More interestingly, PMLR system also elevated population of both anti‐tumor CD8^+^GranzymeB^+^ and CD8^+^IFN‐*γ* ^+^ T cells. Their anti‐tumor efficacy and immunity was further amplified by combining with a‐PD‐L1‐mediated checkpoint‐block in B16F10 tumor.

Improving hypoxia in the tumor microenvironment. Solid tumors have regions of severe hypoxia due to the distorted vessels and the fast tumor proliferation‐initiated imbalance of oxygen supply and consumption.^[^ ^173^, ^174^, ^175^ ^]^ Extensive studies have confirmed the important role of hypoxia in maintaining pro‐tumorigenic M2‐like TAMs. Hypoxia impacts TAMs from two aspects. First, hypoxia‐triggered release of chemoattractants (i.e., CCL2, CCL5, CXCL12, CSF‐1, and VEGF) from tumor cells and non‐tumor cells to enhance TAMs precursor monocytes recruitment. Once the macrophages arrive in hypoxic areas, the corresponding receptors of those chemoattractants were markedly diminished and decreased TAMs mobility to trap them in hypoxic regions. Second, macrophages can directly sense hypoxic environments through hypoxia‐inducible factor 1*α* (HIF1*α*) and become tumor‐supportive M2‐like TAMs.^[^ ^35^, ^174^, ^176^ ^]^ Therefore, improving hypoxia provides great potentials for repolarizing TAMs into anti‐tumor M1 phenotype to enhance anti‐tumor immunity. Manganese dioxide (MnO_2_) nanomaterials can be good candidates for relieving hypoxia due to their intrinsic catalytic property for reaction with the excessive hydrogen peroxide (H_2_O_2_) to generate O_2_ for alleviating hypoxia.^[^ ^177^, ^178^ ^]^ Chang et al. utilized NanoMnSor nanocomposite to deliver MnO_2_ and sorafenib (a first‐line antiangiogenic drug of advanced hepatocellular carcinoma (HCC)) for HCC treatment.^[^ ^179^ ^]^ Since the resistance to sorafenib therapy of HCC tumor is mainly attribute to the hypoxic TME, MnO_2_ could decrease the hypoxia‐triggered tumor‐infiltration of TAMs and promote anti‐tumor M1‐polarization, which ultimately overcame resistance to sorafenib. Chen et al. fabricated core‐shell PLGA nanoparticles loaded with catalase (Cat) and R837.^[^ ^180^ ^]^ These PLGA‐R837@Cat nanoparticles could relieve hypoxia to reprogram immunosuppressive M2‐like TAMs toward anti‐tumor M1 phenotype due to the generation of O_2_ from the degradation of H_2_O_2_ catalyzed by Cat, which led to an enhanced efficacy of tumor radiotherapy. Extensive studies have confirmed that curcumin (Cur) can inhibit HIF1*α* expression and serve as a photosensitizer (PS). Zhang et al. constructed Cur embeded‐core‐satellite upconverting nanoparticles (CSNPs) (Cur‐CSNPs).^[^ ^181^ ^]^ Under NIR irradiation, the Cur‐CSNPs could efficiently overcome O_2_‐dependent PDT‐mediated aggravation of hypoxia via inhibiting HIF1*α* expression, and thus repolarize CD206^+^ M2‐like TAMs into CD86^+^ M1‐like ones. Furthermore, the treatment could reduce primary 4T1 tumor progression and suppress abscopal metastasis via increasing M1 populations, as well as, enhancing CD4^+^ and CD8^+^ T cells infiltration in the distal tumor site.

**FIGURE 8:**
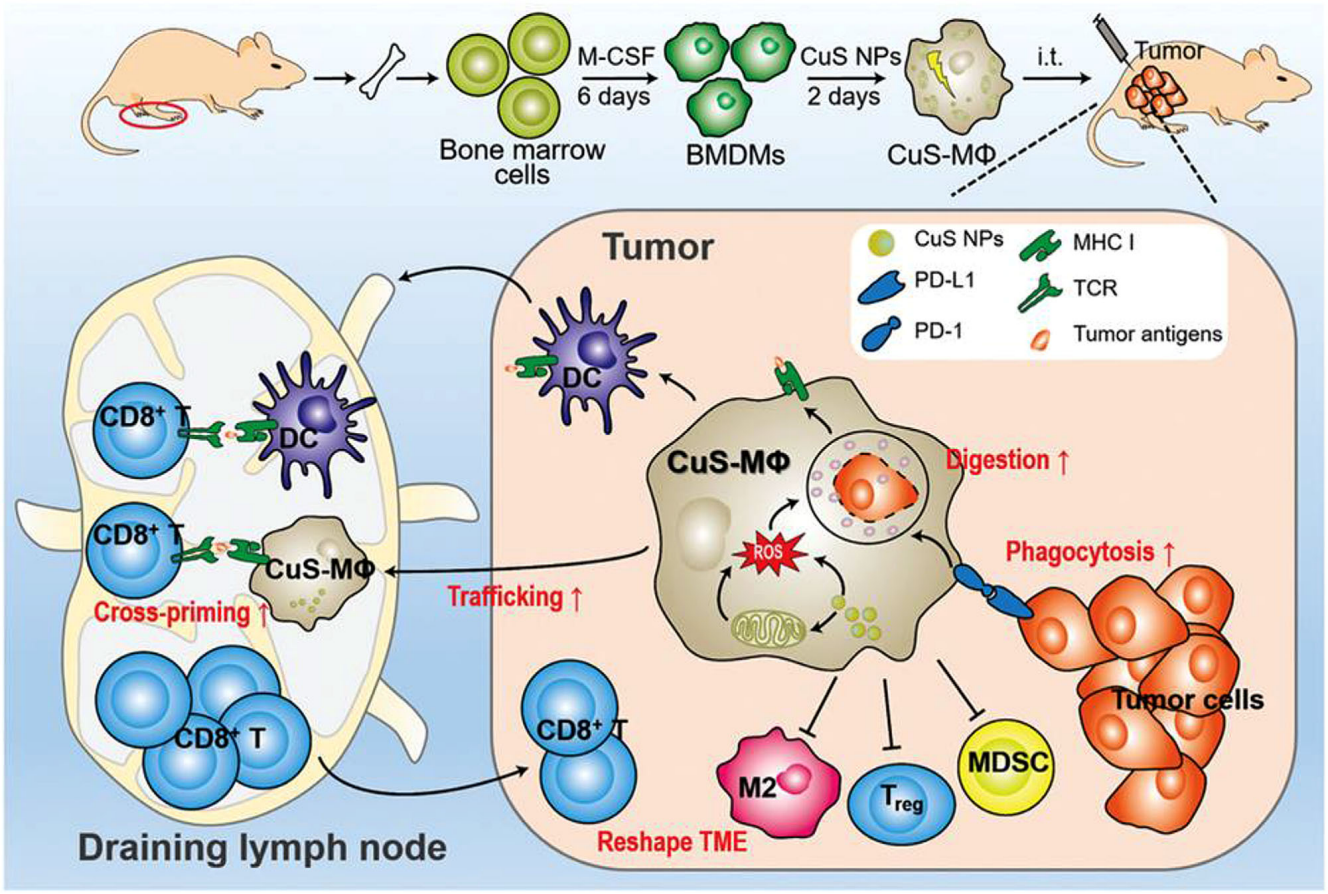
Schematic illustration of adoptive transfer CuS NPs‐repolarized macrophages to treat solid tumor. CuS NPs could polarize BMDMs to M1 phenotype via induce mitochondrial fission‐mediated generation of ROS. The adoptively transferred CuS‐MΦ could not only promote M2‐to‐M1 repolarization, but also enhance tumor‐phagocytosis of macrophages through blocking anti‐phagocytic PD‐1‐PD‐L1 checkpoint, which decreased immunosuppressive tumor‐infiltrating MDSC and Tregs, and enhanced tumor specific CD8^+^ T cell immunity. Reproduced with permission.^[^ ^187^ ^]^ Copyright 2021, John Wiley & Sons

**FIGURE 9:**
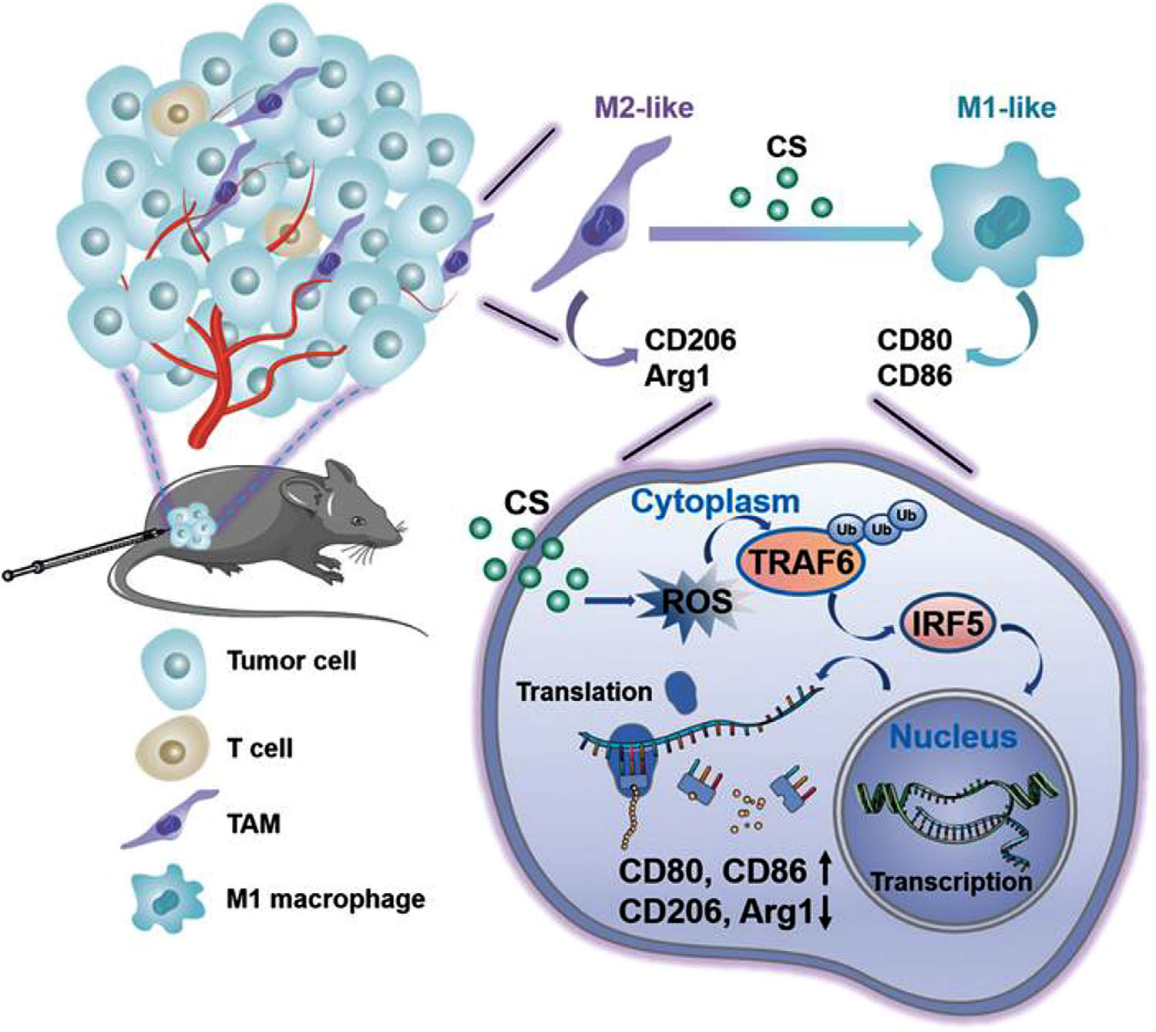
The schematic illustration of CS NPs repolarizing tumor‐associated macrophages (TAMs) toward M1 phenotype to enhance anti‐tumor immunity. CS NPs enhance intracellular ROS level of macrophages to promote TRAF6 auto‐ubiquitination that activates the transcription factor IRF5 to reprogram TAMs toward M1 phenotype. Reproduced with permission.^[^ ^189^ ^]^ Copyright 2021, John Wiley & Sons

Inducing reactive oxygen species generation. ROS, including singlet oxygen (^1^O_2_), OH·, H_2_O_2_, and superoxide anion (O^2−^), are well known for promoting M1‐polarization.^[^ ^23^, ^182^, ^183^, ^184^, ^185^, ^186^ ^]^ Extensive studies have proved that the classical NF‐*κ*B signaling pathway plays critical role in the ROS‐mediated M1‐polarization.^[^ ^110^, ^183^, ^187^, ^188^ ^]^ Therefore, nanoparticles‐mediated ROS generation is promising for promoting M2‐to‐M1 repolarization in the TME. Shi et al. found that the photosensitizers indocyanine green (ICG) and ammonium bicarbonate (NH_4_HCO_3_) co‐loaded in mannose‐modified PEGylated PLGA nanoparticles could generate ROS and reverse pro‐tumor TAMs into anti‐tumor M1 phenotype.^[^ ^183^ ^]^ Moreover, the generated ROS could also increase tumor‐antigen‐presentation and T cell‐activation capability of TAMs in 4T1 tumor. It has been well known that PDT could generate ROS through two mechanisms, in which type I mechanism could produce free radicals and type II mechanism could generate ^1^O_2_.^[^ ^110^ ^]^ To explore the underlying mechanism of ROS generation for TAMs‐repolarization, Yang et al. designed three types of donor‐acceptor (D‐A) structured AIEgen photosensitizers by changing acceptor units. They found that the AIEgen (tTDCR) mediated type I mechanism was the main driver for M1‐polarization.^[^ ^110^ ^]^ Mechanistically, the generated ROS activated NF‐*κ*B signaling pathway through promoting phosphorylation and nuclear translocation NF‐*κ*B, which down‐regulated M2‐related makers and up‐regulated M1‐related makers, leading to great ablation of 4T1 tumor. In another study, Xu et al. developed copper sulfide nanoparticle‐stimulated BMDMs (CuS‐MΦ) for TAMs‐repolarization in mouse melanoma (Figure 8).^[^ ^187^ ^]^ Mechanistically, the CuS nanoparticles (CuS NPs) could produce intracellular ROS in macrophages through Cu ions triggering dynamin‐related protein 1 (Drp1)‐mediated mitochondrial fission, which could be achieved through activation of Mek‐Erk‐Drp1 cascade signal transduction. The generated ROS can persistently facilitate and maintain M1‐polarization by activating IKK‐dependent NF‐*κ*B pathway. The results showed that the CuS‐MΦ could not only enhance tumor‐phagocytosis and digestion of macrophages through down‐regulating expression of surface anti‐phagocytic PD‐1 in vitro, but also polarize M2‐like TAMs into M1 phenotype when they were adoptively transferred into tumor. Furthermore, intratumoral injection of CuS‐MΦ induced remarkably anti‐tumor immunity via decreasing other immunosuppressive cell subsets including Treg cells and MDSCs, increasing tumor‐infiltrating IFN‐*γ*‐positive CD8^+^ T cells and Granzyme B‐positive CD8^+^ T cells, as well as activating DCs. Zheng et al. found that ultra‐small copper selenide nanoparticles (Cu_2‐_ *_x_* Se, also denoted as CS NPs) could significantly promote M2‐to‐M1 polarization and obviously inhibit the progression and recurrence of B16F10 tumor via a novel ROS‐mediated macrophage polarization mechanism (Figure 9).^[^ ^189^ ^]^ The CS NPs could robustly enhance ROS level in macrophages, which facilitated the auto‐ubiquitination of TRAF6 and then induce TRAF6 downstream factor IRF5 activation. Finally, the IRF5 downstream gene IL‐23 were significantly up‐regulated. More importantly, the ROS‐TRAF6‐IRF5‐IL‐23 signaling pathway is completely different from the traditional ROS‐NF‐*κ*B‐iNOS pathway. Zou et al. prepared an artificial NK cell (aNK), which was formed by coating perfluorohexane (PFC) and glucose oxidase (GOX) with red blood cell membrane (RBCM).^[^ ^188^ ^]^ The aNK could directly kill tumor cells by decomposing glucose to generate H_2_O_2_ under the catalysis of GOX, in which the catalytic reaction was also strengthened by oxygen‐carried PFC. As a stable ROS, H_2_O_2_ could significantly re‐model tumor‐supportive TAMs to anti‐tumor CD80^+^CD86^+^ M1 phenotype via down‐regulating M2‐associated CD206 marker and up‐regulating M1‐assoiated‐CD86, CD80, and MHCII markers. More importantly, the aNK system also attracted CD8^+^ T cells infiltration, resulting in dramatical reduction of 4T1 tumor.

Reprograming tumor‐associated macrophages via nanoparticles‐based photoimmunotherapy. PDT has been widely used to elicit ROS‐mediated M2‐to‐M1 repolarization as mentioned above, PTT has been also demonstrated to induce M1‐polarization. He et al. found that RBC membrane‐camouflaged 2D MoSe_2_ nanosheets (RBC‐MoSe_2_)‐mediated PTT could obviously decrease M2‐related Arg1 and CD206 mRNA levels and increase tumor‐inhibiting M1‐related TNF‐*α* and iNOS2 mRNA levels.^[^ ^190^ ^]^ Mechanically, the PTT could ablate tumors and then release tumor‐associated antigens, resulting in enhanced specific tumor antigen‐presentation and CD8^+^ T cell activation. The activated CD8^+^ T cells secreted large amount of IFN‐*γ* to induce TAMs‐repolarization.

In addition, light itself can also regulate macrophage phenotypes. For example, the NIR light can temporally regulate the intracellular calcium levels to control macrophage phenotypes.^[^ ^191^ ^]^ Kang et al. constructed mesoporous silica‐coated upconversion nanoparticles (UCNP@mSiO_2_) and modified with a photocleavable Arg‐Gly‐Asp (RGD) peptide bearing molecular cap via cyclodextrin‐adamantine coupling, which could response to the NIR irradiation for releasing calcium regulators to supply or eliminate calcium ions. Compared to control experiments, Cap‐DMNP‐UCNP@mSiO_2_‐mediated intracellular calcium elevation promoted M1‐polarization and Cap‐BAPTA‐UCNP@mSiO_2_‐mediated intracellular calcium depletion facilitated M2‐polarization. More importantly, the skin tissue‐mediated remote manipulation of macrophage polarization suggested the potential of application of photo‐responsive nanocarrier‐mediated intracellular calcium‐regulation for TAMs‐polarization in vivo.

### 3.4 Regulating macrophage‐mediated tumor‐phagocytosis

As an important type of professional phagocytes in the innate immune system, macrophages act as a first line nonspecific recognition and defense to against invasion of pathogens and malignancies.^[^ ^192^, ^193^ ^]^ To evade the immune surveillance, living tumor cells can mimic the intrinsic properties of normal cells to resist the phagocytosis by overexpressing anti‐phagocytotic proteins, which can interact with their corresponding receptors on macrophages and send “don't eat me” signals to macrophages.^[^ ^194^, ^195^, ^196^ ^]^ In contrast, the apoptotic cells within TME can be rapidly identified and cleared by macrophages via exposing “find me” signals and “eat me” signals, in which the process was termed efferocytosis, resulting in “immunologically silent” response characterized by an anti‐inflammatory state in the TME and facilitating tumor immune escape.^[^ ^16^, ^197^ ^]^ Thus, modulating tumor cell‐phagocytosis of macrophages holds great potentials for tumor immunotherapy and could be achieved by targeting phagocytosis checkpoints in live tumor cells or inhibiting macrophage‐mediated efferocytosis to apoptotic tumor cells in TME.

#### 3.4.1 Targeting phagocytotic checkpoints against living tumor cells

Up to now, four phagocytotic checkpoints have been well‐identified including CD47‐SIRP*α*, PD‐L1‐PD‐1, major histocompatibility class I complex (MHC‐I)‐leukocyte immunoglobulin‐like receptor 1 (LILRB1), and CD24‐sialic‐acid‐binding Ig‐like lectin 10 (Siglec‐10) axes. Tumor cells overexpress anti‐phagocytic ligands CD47, PD‐L1 MHC‐I, and CD24, and can interact with their corresponding receptors SIRP*α*, PD‐1, LILRB1, and Siglec‐10 on the macrophages and send “don't eat me” signals to evade phagocytic clearance by macrophages.

##### Phagocytotic checkpoints

CD47 is a transmembrane‐bound protein expressed by all normal cells throughout the body, particularly overexpressed by young red blood cells and multiple tumor cells.^[^ ^198^, ^199^, ^200^ ^]^ Engagement of CD47 with SIRP*α* initiates the phosphorylation of two tyrosine residues of SIRP*α* cytoplasmic ITIM, leading to subsequent recruitment and activation of SH2‐domain‐containing phosphatase1 (SHP1) and 2 (SHP2). This process consequently results in dephosphorylation of myosin IIA, and thereby suppresses cytoskeleton rearrangement and prevents phagocytosis.^[^ ^198^, ^201^, ^202^ ^]^ Therefore, CD47‐SIRP*α* interactions make a wide range of tumor cells transmit “don't eat me” signals to macrophages, thereby promotes tumor immunological escape.^[^ ^196^, ^200^, ^203^ ^]^ To date, various agents for blocking the CD47‐SIRP*α* interactions have been developed, including antibodies, inhibitors, siRNAs, and analogs, etc.^[^ ^17^, ^36^ ^]^

PD‐1 is a transmembrane protein and owns two ligands, PD‐L1 and PD‐L2. The interactions of PD‐1 expressed on CD8^+^ T cells with its ligand PD‐L1 overexpressed on tumor cells paly critical role in T cell anergia or exhaustion. Therefore, PD‐L1‐PD‐1 axis was initially identified as a T cell immunocheckpoint and extensively manipulated to potentiate activation of tumor‐specific T cell immunity.^[^ ^204^, ^205^, ^206^ ^]^ However, PD‐L1‐PD‐1 axis is also an important phagocytotic checkpoint beyond adaptive checkpoint, which can inhibit tumor‐phagocytosis capability of TAMs.^[^ ^207^ ^]^ In this study, Gordon et al. found that PD‐1^+^ TAMs from mouse and human colorectal tumors were M2 phenotype, and PD‐1^–^ TAMs were M1 phenotype. These PD‐1^–^ TAMs exhibited much higher phagocytic capability of CT26 tumor cells than M2 PD‐1^+^ TAMs. More importantly, PD‐1 was mainly expressed on M2‐like TAMs rather than circulating monocytes or splenic macrophages, PD‐1^+^ TAMs were mainly sourced from circulating leukocytes, and accumulated within the TME with stage of tumor.

Blocking PD‐1‐PD‐L1 axis by anti‐PD‐1 antibody or a PD‐L1 blocker increased tumor‐engulfment and reduced tumor progression in immunocompromised mice lacking the adaptive immune system but retaining functional macrophages, which suggests that PD‐1‐PD‐L1 interactions can inhibit tumor‐phagocytosis in a macrophage‐dependent manner. However, it is worthy noted that PD‐1 is also highly expressed on other anti‐tumor immune cells including B cells,^[^ ^208^, ^209^ ^]^ NK cells,^[^ ^210^, ^211^ ^]^ and DCs,^[^ ^212^, ^213^ ^]^ as well as, overexpressed on immunosuppressive myeloid cells.^[^ ^214^ ^]^ Therefore, the response of patients to inhibition of PD‐1‐PD‐L1 interactions may derive from action of multiple immune cells, and the clear mechanism of PD‐1 mediated blunt tumor‐phagocytosis of macrophages needs to be elucidated.

MHC‐I, a heterodimer of heavy *α*‐chain and *β*2‐microglobulin (B2M), exists in many nucleated cells, which play functions of presenting antigens to T cells.^[^ ^215^ ^]^ Barkal et al. found that MHC‐I expressed on tumor cells played a critical role in resisting macrophages‐mediated phagocytosis via the interaction of its B2M subunit with LILRB1 on macrophages.^[^ ^216^ ^]^ Genetically blocking MHC‐I‐LILRB1 axis could elicit tumor‐phagocytosis of macrophages in vitro and in vivo. Furthermore, the NSG mice lacking all MHC‐I‐sensitive immune cell subsets (e.g., T cells or NK cells) showed obvious tumor‐phagocytosis of macrophages in vivo, suggesting a macrophage‐MHC‐I mediated anti‐phagocytic effect. More importantly, blocking MHC‐I‐LILRB1 signaling pathway also contributes to NK cell‐mediated anti‐tumor response.^[^ ^217^ ^]^ Therefore, the tumor‐inhibiting response initiated by blocking MHC‐I‐LILRB1 axis may be a synergetic effect of macrophages and NK cells. Identification of precise mechanism of MHC‐I‐LILRB1‐mediated inhibition or development of TAMs‐targeting MHC‐I‐LILRB1‐blocking strategies is needed for overcoming tumor escape from macrophage‐mediated phagocytosis.

CD24, a glycosylphosphatidylinositol‐anchored membrane glycoprotein,^[^ ^218^, ^219^ ^]^ is another newly discovered “don't eat me” signal, which is overexpressed on several types of tumor cells, and interacts with Siglec‐10 on TAMs to facilitate tumor evasion from TAMs‐mediated phagocytosis.^[^ ^218^ ^]^ In this study, Barkal et al. found that Siglec‐10 was mainly overexpressed on TGF*β*1‐ and IL‐10 induced M2‐like macrophages rather than unstimulated human donor‐derived M0‐like macrophages. Genetic depletion of either CD24 or Siglec‐10 or blocking the CD24‐Siglec‐10 signaling with monoclonal antibodies remarkably enhanced TAMs‐mediated tumor‐engulfment in human tumor model, which suggests that M2‐like macrophages also could be manipulated to inhibit tumor progression by enhancing their tumor‐phagocytosis activity.

##### Intervening phagocytotic checkpoints by nanoparticles

**FIGURE 10:**
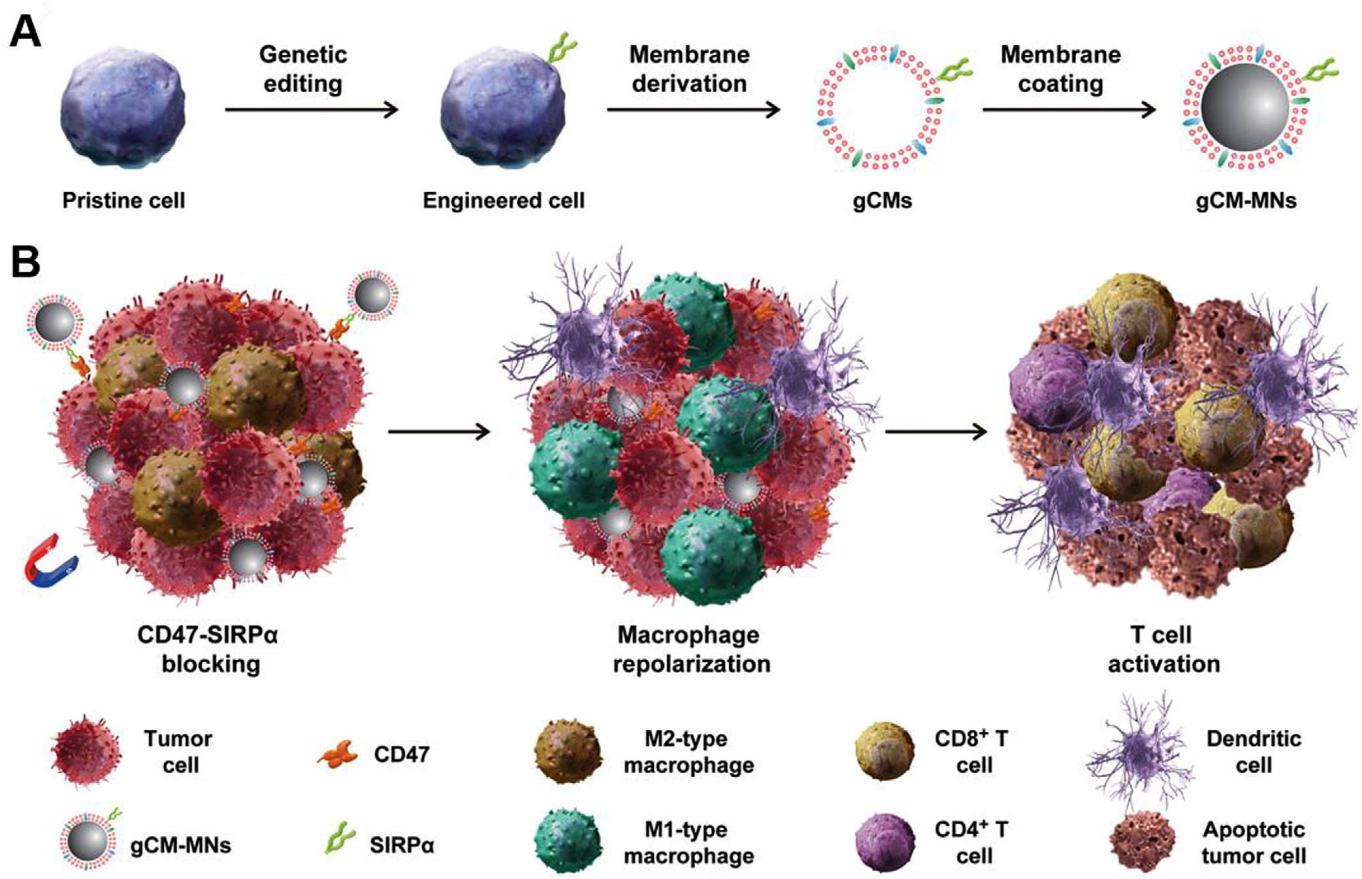
Schematic illustration of genetically engineered cell‐membrane‐coated magnetic nanoparticles (gCM‐MNs) blocking CD‐47‐SIRP*α* anti‐phagocytic checkpoint for tumor immunotherapy. (A) The fabrication of gCM‐MNs, including the gene edition of cells to overexpress SIRP*α* variants on the membrane, the isolation of cell membrane, and the coating of magnetic nanoparticles (MNs) with membrane. (B) The resultant gCM‐MNs could efficiently accumulate into tumor site upon the external magnetic field guidance, and inhibit CD47‐ SIRP*α* “don't eat me” signaling, leading to effective M2‐to‐M1 repolarization, enhanced tumor‐phagocytosis of macrophages and potent tumor‐specific T cell immunity. Reproduced with permission.^[^ ^220^ ^]^ Copyright 2020, John Wiley & Sons

Up to now, the CD47‐SIRP*α* signaling is the most studied anti‐phagocytic signal in tumor.^[^ ^13^ ^]^ Rao et al. used gene‐edited strategy to make cell overexpress SIRP*α* variants (Figure 10).^[^ ^220^ ^]^ The SIRP*α* variants possess remarkable affinity to effectively block CD47‐SIRP*α* axis, thereby enhancing the phagocytosis of cancer cells by macrophages. Combining with the core‐mediated M2‐like TAMs‐repolarization, the gCM‐MNs could significantly prevent tumor recurrence and metastasis in malignant melanoma model. Unfortunately, blocking CD47‐SIRP*α* is not enough when the pro‐phagocytic molecule calreticulin (CALR) on tumor cells is low. Therefore, Zhang et al. developed a pro‐phagocytic nanoparticle (SNPA_CALR&aCD47_) system to co‐deliver anti‐CD47 and CALR.^[^ ^221^ ^]^ Anti‐CD47 antibody could block CD47‐SIRP*α* to promote the macrophages to “eat” tumor cells, meanwhile the CALR can amplify the tumor‐phagocytosis of macrophages and eventually enhance the macrophage‐based cancer immunotherapy.

There are limited studies on improving the tumor‐phagocytosis capability of macrophages by blocking PD‐L1‐PD‐1, MHC‐I‐LILRB1, and CD24‐Siglec‐10 phagocytic immune checkpoint. Since CD47 is expressed by all cells throughout the body, and SIRP*α* is only expressed on the neuronal cells and myeloid cells,^[^ ^36^ ^]^ developing novel strategies to against SIRP*α* holds much more superiority to enhance tumor‐phagocytosis of macrophages and reduce side effects.

#### 3.4.2 Regulating macrophage‐mediated efferocytosis of dying/apoptotic cells

Efferocytosis, a clearing process of apoptotic cells, can be performed by many types of phagocytes, in which macrophages are major professional phagocytes to scavenge dying and apoptotic cells.^[^ ^17^, ^222^ ^]^ Mechanistically, the efferocytosis is carefully orchestrated by two steps.^[^ ^16^ ^]^ First, the apoptotic cells release “find me” signals to attract phagocytes to their site. Second, the attracted phagocytes can recognize “eat‐me” signals through their specific phagocytic receptors, and subsequently engulf and process apoptotic cells to inhibit the inflammatory responses.^[^ ^16^, ^223^ ^]^ Tumor cells can be killed by specific cell‐death inducers within a tumor milieu, for example, chemotherapy, radiotherapy, and PDT could induce apoptosis of abundant tumor cells. Rapid elimination of these dying/apoptotic cells by phagocytes in the TME could trigger “immunologically silent” condition, and facilitate tumor immune escape and progression.^[^ ^20^, ^224^ ^]^ In contrast, blocking efferocytosis of dying/apoptotic tumor cells would further undergo the secondary necrosis, which could yield many tumor‐associated antigen epitopes and release damage‐associated molecular patterns (DAMPs) to reverse immunogenic silence into immunogenic activation, and subsequently elicit adaptive anti‐tumor activity.^[^ ^17^, ^20^ ^]^ Since macrophages are major professional phagocytes, and main immunosuppressive component in the TME, manipulating macrophage‐mediated efferocytosis of dying/apoptotic cells holds great promise for inhibiting tumor progression and eliciting potent tumor‐specific immunity.

##### Find me and eat me signals

Up to now, four “find me” signals have been identified on the dying/apoptotic cells, including lipid lysophosphatidylcholine (LPC), nucleotides (e.g., adenosine triphosphate (ATP) and uridine triphosphate (UTP)),sphingosine 1‐phosphate (S1P), and the fractalkine C‐X3‐C motif ligand 1 (CX3CL1). They can promote macrophage chemotaxis by interacting with the macrophage receptors G protein‐coupled receptor G2A, P2Y purinoceptors, S1PR1‐S1PR5, and CX3CR1, receptively.^[^ ^17^, ^197^, ^222^ ^]^

Typically, PtdSer is the most well‐known “eat me” signal for attracting phagocytes, which exists on the outer leaflet of the dying/apoptotic cells membrane.^[^ ^225^, ^226^ ^]^ It can be recognized by the specific receptors expressed by phagocytes, especially macrophages, to initiate phagocytosis and processing of dying/apoptotic cells and trigger inhibition of pro‐inflammatory responses.^[^ ^16^ ^]^ There are two kinds of PtdSer recognition receptors, which can be classified into direct and indirect binding receptors of PtdSer ligand.^[^ ^197^ ^]^ The direct PtdSer‐binding receptors can directly recognize and interact with PtdSer, including T cell immunoglobulin and mucin (TIM) protein family (e.g., TIM‐1, TIM‐3, and TIM‐4), brain‐specific angiogenesis inhibitor 1 (BAI1), stabilin 2, and C300b.^[^ ^197^, ^222^ ^]^ Among the TIM family, TIM4 is strongly associated with macrophage‐mediated efferocytosis (particularly peritoneal macrophages rather than alveolar macrophages and microglia). TIM1 and TIM3 are primarily expressed in Th2 and Th1 cells, as well as, plasmacytoid DCs.^[^ ^222^ ^]^ Additionally, TIM‐4‐mediated phagocytosis of apoptotic cells through cooperation with MerTK rather than its cytoplasmic and transmembrane regions, which suggests that TIM‐4 only exerts tethering function.^[^ ^17^, ^197^ ^]^ Alternatively, indirect PtdSer‐binding receptors bind with PtdSer in a bridging molecule‐dependent manner. These bridging molecules include milk fat globule EGF factor 8 (MFGE8), and growth arrest‐specific Gas6 and protein S (ProS1), in which MFGE8 can bridge PtdSer and integrins *α*v*β3* and *α*v*β5*, while Gas6 and ProS1 bridge PtdSer and TAM receptors (MerTK, Axl, and Tyro3).^[^ ^17^ ^]^ Notably, Gas6 recognizes and binds to all three types of TAM receptors, whereas ProS1 can only link to MerTK and Tyro3.^[^ ^226^, ^227^ ^]^ Following apoptotic cell recognition and tethering, cytoskeletal rearrangements induced phagosome formation to completely phagocyte the apoptotic cell.^[^ ^17^, ^19^ ^]^

Among the PtdSer (“eat me” signal) recognition system, the TAM receptors and their activating ligands are extensively expressed in macrophages, which are well‐studied in macrophage‐mediated efferocytosis in the TME.^[^ ^17^, ^222^ ^]^ Among three TAM receptors involved in macrophage‐mediated efferocytosis, MerTK is the crucial one.^[^ ^19^ ^]^ Although Axl and Tyro3 participate in mediating efferocytosis in macrophages, they may dominantly mediate efferocytosis in DCs. In addition, the role of Tyro3 remains largely unclear.^[^ ^19^, ^222^ ^]^ MerTK‐mediated efferocytosis in macrophages has been involved in inhibiting M1‐polarization and facilitating macrophages to polarize into anti‐inflammatory M2 phenotype.^[^ ^19^, ^226^ ^]^ For examples, MerTK activation has been confirmed to inhibit generation of pro‐inflammatory cytokines TNF‐*α* and IL‐6 in human monocytes/macrophages and block TNF‐*α* production in LPS‐stimulated mouse macrophages.^[^ ^228^, ^229^ ^]^ Similarly, Gas6‐MerTK signaling could up‐regulate the expression of M2‐related VEGF and Arg1 makers in RAW 264.7 macrophages. The MerTK‐expressing TAMs could transfer into pro‐tumor M2 phenotype upon stimulation by apoptotic materials.^[^ ^226^ ^]^ More importantly, M2‐like macrophages express much higher MerTK and exhibit stronger capability of efferocytosis than M1‐like macrophages.^[^ ^230^ ^]^ Therefore, blocking PtdSer exposed by dying/apoptotic tumor cells and MerTK activation to blunt macrophage‐mediated efferocytosis could be a promising method for alleviating the immunosuppressive TME via reversing the anti‐inflammatory M2‐like TAMs.

##### Engineering nanoparticles to inhibit macrophage‐mediated efferocytosis

Annexin A5 (ANX5) protein, a member of annexin family, can bind and block PtdSer exposed by apoptotic cells. On this basis, Li et al. fabricated diselenide‐bridged hollow mesoporous organosilica nanoparticles for A5 delivery (HMSeN‐ANX5@HOMV).^[^ ^20^ ^]^ HMSeN@HOMV under laser irradiation (LI) could significantly induce PtdSer exposure of apoptotic 4T1 cells to greatly eliminate dying tumor cells by macrophages (BMDMs)‐mediated phagocytosis. In contrast, HMSeN‐ANX5@HOMV could substantially abate and reverse tumor‐phagocytosis by macrophages and facilitate tumor apoptosis to secondary necrosis under LI stimulation. In vivo study shows that HMSeN@HOMV plus LI could induce burst release of ANX5 to block PtdSer exposure on the membrane of dying 4T1 tumor cells, and thus reverse anti‐inflammatory M2‐like TAMs toward pro‐inflammatory M1 phenotype, which also led to potent inhibition of immunosuppressive MDSCs and Tregs, as well as, activation of DCs and tumor‐specific T cell immunity.

Inhibiting phagocytic receptor MerTK to block TAMs‐mediated efferocytosis is another efficient strategy for tumor therapy. Zhou et al. designed a functional antibody to selectively inhibit MerTK rather than Tyro3 and Axl or other surface proteins on macrophages, which greatly suppressed MerTK‐dependent engulfment of dying cells by TAMs and also elicited potent STING activation in macrophages.^[^ ^231^ ^]^ In addition, Su et al. found that MRX‐2843, an MerTK inhibitor, could decrease immunosuppressive M2‐like glioblastoma‐associated macrophages and microglia (GAMs).^[^ ^230^ ^]^ Therefore, development of nanotechnology for targeting inhibition of MerTK on macrophages is necessary for further improving MerTK‐dependent anti‐tumor therapy.

It is very hard to develop inhibitors to specifically interact with one of the TAM receptors, because all TAM receptors possess similar structure.^[^ ^17^ ^]^ Importantly, in addition to macrophages, various tumor cells (i.e., ovarian tumor, non‐small cell lung tumor, colorectal cancer, breast tumor, and melanoma) also overexpress MerTK and Axl, as well as, their ligands, which are well correlated with their poor prognosis.^[^ ^226^, ^232^ ^]^ MerTK and Axl also play important roles in inducing DCs‐mediated immunosuppressive state.^[^ ^222^ ^]^ Therefore, blocking MerTK and Axl in the TME could cause a superimposed anti‐tumor effect. Sometimes, the inhibitors of one TAM receptor may synergistically inhibit multiple receptors on different immune cells or tumor cells, resulting in an addition anti‐efferocytosis effect. It is also worthy noted that MerTK is also expressed on CD4^+^ and CD8^+^ T cells, which plays costimulatory role in promoting activation and function of T cells.^[^ ^232^ ^]^ Therefore, blocking macrophage‐mediated MerTK signaling of efferocytosis would impair functions of tumor‐specific T cells.^[^ ^232^ ^]^ Thus, development of nanotechnology for targeted inhibition of macrophages‐mediated efferocytosis is indeed necessary. Selective blockade of PtdSer on tumor cells could avoid side effects caused by indiscriminate inhibition of all PtdSer‐mediated efferocytosis in the whole body.

## 4 ENGINEERING MACROPHAGES AS DRUG DELIVERY VEHICLES

Macrophages and their precursor monocytes possess inherent capabilities of homing to tumor site, because they can cross multiple biological barriers such as the tumor core or blood brain barrier (BBB) via responding to various chemoattractants (i.e., CSF‐1, VEGF, platelet‐derived growth factor, CCL2, CCL5, CCL7, CCL8, CCL22, CXCL8, CXCL12, etc.) and hypoxic conditions in the TME.^[^ ^5^, ^28^, ^29^ ^]^ In addition, macrophages have natural capability of phagocyting tumor cells and evading unwanted immune reactions.^[^ ^233^ ^]^ These unique properties of macrophages have attracted much attention and drive researchers to engineer macrophages and macrophage‐derived components (i.e., macrophage membrane and macrophage‐derived extracellular vesicles (EVs)) to treat tumors.^[^ ^5^, ^28^, ^36^ ^]^

### 4.1 Macrophages as “Trojan Horses”

#### 4.1.1 Encapsulating nanoparticles and drugs into macrophages

**FIGURE 11:**
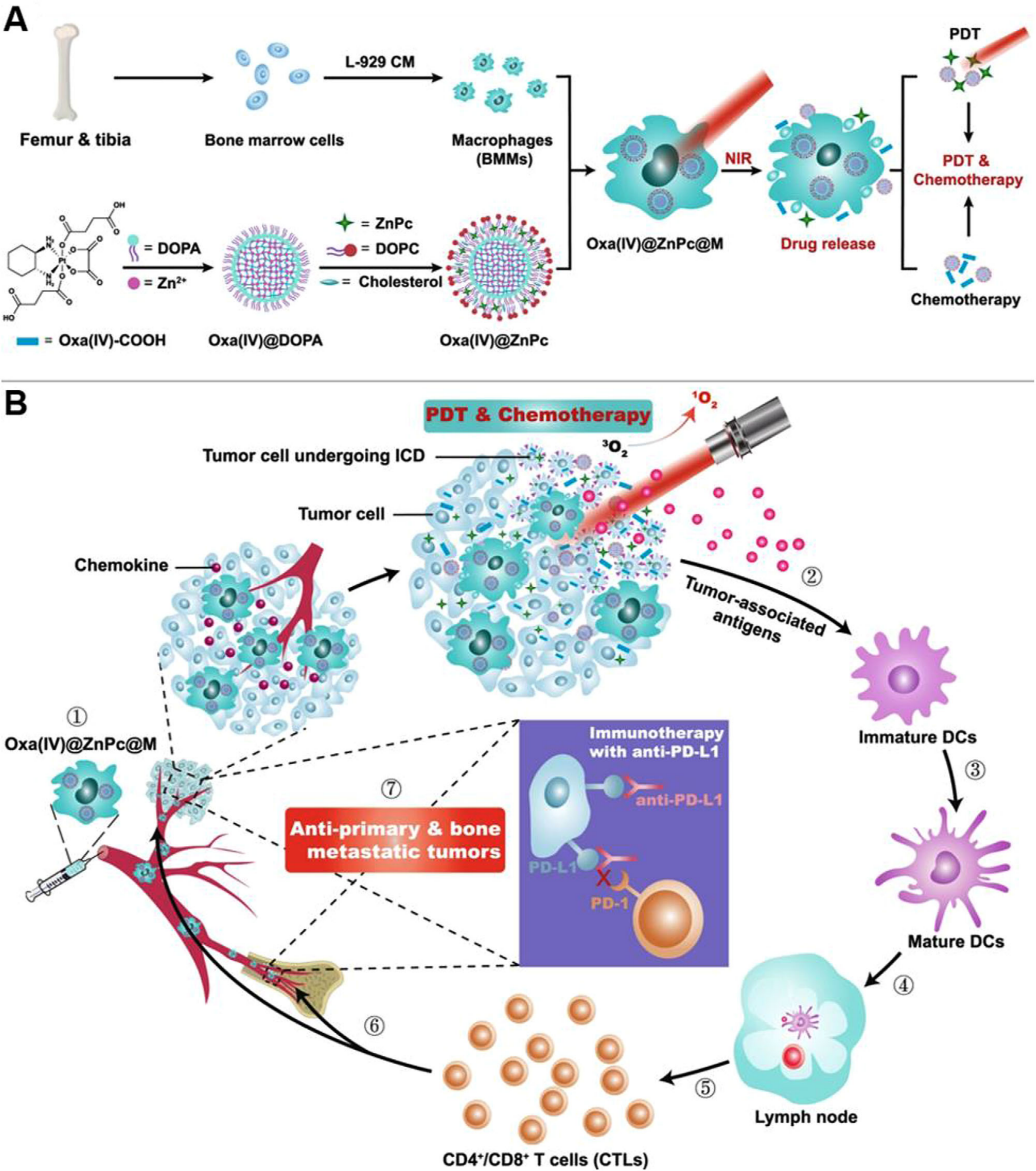
Schematic illustration of engineering living macrophages for drug delivery to treat solid tumor. (A) The synthesis of Oxa(IV)@ZnPc@M. (B) Oxa(IV)@ZnPc@M displayed an pro‐inflammatory M1 phenotype polarization and could efficiently target primary and bone metastatic tumor. The combined chemo‐photodynamic therapy induced ICD to release tumor‐associated antigens and elicit DCs maturation and effective anti‐tumor immunity, which could significantly suppress primary and bone metastatic tumor progression by combining with anti‐PD‐L1 immunotherapy. Reproduced with permission.^[^ ^233^ ^]^ Copyright 2021, Springer Nature

Macrophages are extensively engineered as “Trojan Horses” for targeted delivery of their internalized drugs into tumor site. Direct encapsulation of anti‐tumor drugs into macrophages could rapidly kill macrophages due to the severe cytotoxicity of these drugs.^[^ ^36^ ^]^ Alternatively, indirect encapsulation of drug‐loaded nanoparticles or nanoparticles are more effective ways. Wayne et al. used macrophages to phagocytose calcium integrin binding protein‐1 (CIB1)‐siRNA lipoplexes and horizontally transferred this payload into the orthotopic MDA‐MB‐468 human breast tumor, resulting in down‐regulation of proliferation and survival‐promoting CIB1 and KI67 gene expressions in MDA‐MB‐468 tumor cells, thereby inducing great suppression of tumor growth.^[^ ^234^ ^]^ To avoid the premature release of drugs and toxicity to host cells, the NIR‐responsive macrophage‐based delivery system was developed. Huang et al. genetically engineered macrophage to express a non‐secreted form of EGFP‐TNF‐*α* fusion protein and encapsulated a NIR‐responsive heat‐nanogenerators (HIMs) into macrophages.^[^ ^235^ ^]^ Due to the intrinsic tumor tropism of macrophages and NIR‐responsive capability of HIMs, the resultant HIMs@eM^ET^ could actively target tumor site and spatiotemporally control the release of non‐secreted TNF‐*α*, leading to selective and remarkable toxicity to tumors. Similarly, the engineered NIR‐activatable drug vectors Oxa(IV)@ZnPc@M, which were fabricated by encapsulating oxaliplatin prodrug and photosensitizer into macrophages, could also potentiate chemo/photo immunotherapy of primary and bone metastatic tumors. The Oxa(IV)@ZnPc@M could also induce effective M1‐polarization and significant ICD to generate “in situ vaccines,” and improve anti‐PD‐L1 immunotherapy (Figure 11).^[^ ^233^ ^]^ Sun et al. prepared smart macrophage vehicles through phagocytosis of DOX‐loaded MnO_2_ shell wrapped‐mesoporous carbon nanospheres.^[^ ^236^ ^]^ The resultant macrophage vehicles could exhibit NIR control release performance to avoid the side effects of unwanted released drug. The released MnO_2_ nanoparticles could react with H_2_O_2_ to generate O_2_ in the TME, leading to the controlled release of DOX and enhanced chemo/chemodynamic synergistic therapy. To further improve the tumor tropism, Nguyen et al. fabricated dual‐targeting trojanized macrophage‐based drug carriers by packaging the paclitaxel (PTX)‐encapsulated magnetic liposomes (PTX‐MLPs) into J774A1 macrophages.^[^ ^237^ ^]^ These macrophage‐based carriers could effectively target tumor site through both external electromagnetic field and macrophage‐initiated tumor‐homing property. More interestingly, Zhang et al. used macrophages to internalize dox‐silica nanocomplexes, in which the drugs were electrostatically bound with silica to avoid their burst release. The resultant macrophage carriers could control drug‐release into tumor via a two‐phased drug release profile,^[^ ^29^ ^]^ and the U87MG xenograft tumors were significantly inhibited after intravenous (i.v.) injection of DOX‐loaded macrophages.

Modifying macrophages with enzyme‐responsive peptides or genetically engineering macrophages can further enhance tumor‐targeting capability and anti‐tumor performance of macrophage‐based delivery system. Cao et al. prepared an intelligent legumain protease‐responsive macrophage‐based delivery system (LD‐MDS) via anchoring a legumain‐specific propeptide of melittin (legM) and cytotoxic soravtansine (DM4) prodrug onto the membrane of macrophages.^[^ ^238^ ^]^ The resultant LD‐MDS could response to legumain protease and subsequently transform into DM4‐loaded exosome‐like nanovesicles (DENs), which can be internalized by metastatic 4T1 tumor cells. More interestingly, the damaged 4T1 cells could also release secondary nanovesicles and free drugs to disrupt neighboring tumor cells, resulting in great inhibition of lung metastasis.

#### 4.1.2 Macrophage backpacks

Another effective strategy for engineering macrophages as “Trojan Horses” is to anchor drug‐loaded backpacks (BPs) onto the surface of macrophages, which can prevent the payloads from endosomal degradation and enhance efficiency of tumor‐targeted drug delivery.^[^ ^5^, ^239^ ^]^ Cellular backpacks can serve as drug repositories. They are micron‐scale patches with a few hundred nanometers in thickness, in which the cell adhesive layer can be constructed by layer‐by‐layer (LbL) assembly.^[^ ^239^, ^240^ ^]^ One prerequisite is that the BPs should not be internalized by macrophages and affect macrophage function, because macrophages are highly phagocytic and plastic.^[^ ^5^ ^]^ It is worthy noted that the adoptively transferred macrophages are susceptible to polarization into pro‐tumor M2 phenotype by immunosuppressive factors in the TME.^[^ ^187^ ^]^ To solve this problem, Shields et al. adhered the cell‐adhesive layer‐ stabilized IFN‐*γ* BPs to the surface of macrophage (BMDM), which could sustainably promote anti‐tumor M1‐polarization even in an immunosuppressive TME, resulting in robust regression of breast tumor and metastasis.^[^ ^240^ ^]^ More importantly, the resultant IFN‐*γ*‐macrophage‐BPs could evade phagocytosis of macrophages for several days and maintain the activity of IFN‐*γ* for a long time, which suggests the great potential of engineering TAMs‐repolarization agents‐loaded macrophage‐BPs to enhance anti‐tumor therapy.

It should be noted that, M1‐like macrophage‐based drug delivery system can not only act as a carrier, but also maintain their intrinsic anticancer function. For example, the BMDMs stimulated by CuS nanoparticle (CuS‐MΦ) could not only maintain M1 phenotype with enhanced tumor‐phagocytosis and digestion capabilities, but also promote M2‐to‐M1 repolarization to enhance anti‐tumor immune response, which resulted in robust tumor suppression^[^ ^187^ ^]^ Similarly, the IFN‐*γ*‐macrophage‐BPs could not only release cytokines to continuously stimulate polarization of M2‐like macrophages into anti‐tumor M1 phenotype, but also maintain their anti‐tumor M1 phenotype in vivo.^[^ ^240^ ^]^ Therefore, M1‐like macrophage‐based drug delivery system holds great potential to take dual actions of tumor‐targeted drug delivery and tumor inhibition.

### 4.2 Macrophage membrane

Macrophage membrane coated nanoparticles have been extensively and successfully applied to enhance cell‐cell adhesion for tumor‐targeting and prolong their circulation in the blood stream.^[^ ^103^, ^241^ ^]^ Zhang et al. fabricated macrophage membrane‐coated nanoparticles (cskc‐PPiP/PTX@Ma) with excellent tumor‐homing capability due to the intrinsic property of macrophage membrane.^[^ ^242^ ^]^ The resultant formulations displayed pH‐responsive property for step‐by‐step controlled release of drugs. Since the metastatic tumors are characterized by poor vasculature and angiogenic dormancy, the efficacy of EPR‐depend nanoparticle delivery is limited.^[^ ^31^, ^243^ ^]^ Cao et al. found that coating pH‐sensitive emtansine‐liposome with macrophage membranes (RAW 264.7) could efficiently enhance their cellular accumulation in the metastatic sites of 4T1 tumor.^[^ ^31^ ^]^ Mechanically, *α*4 and *β*1 integrins overexpressed on RAW 264.7 cells could interact with vascular cell adhesion molecule‐1 (VCAM‐1) overexpressed on 4T1 cell membrane, and endow macrophage membrane with inherent homing capability to metastatic 4T1 tumor. In addition, macrophage membrane could also be engineered to further improve their tumor‐targeting capability. Zhang et al. utilized a tumor targeting peptide RGD to modify macrophage (J774A.1) membrane and packed magnetic nanocluster (MNC):siRNA complex. The modified nanocomplex could successfully achieve tumor‐targeting siRNA delivery by multiple effects, including stealth effect of macrophage membrane, magnetic accumulation, and tumor‐targeting capability of RGD.^[^ ^244^ ^]^ The complex could significantly suppress human breast tumor (MCF‐7 xenografts).

**FIGURE 12:**
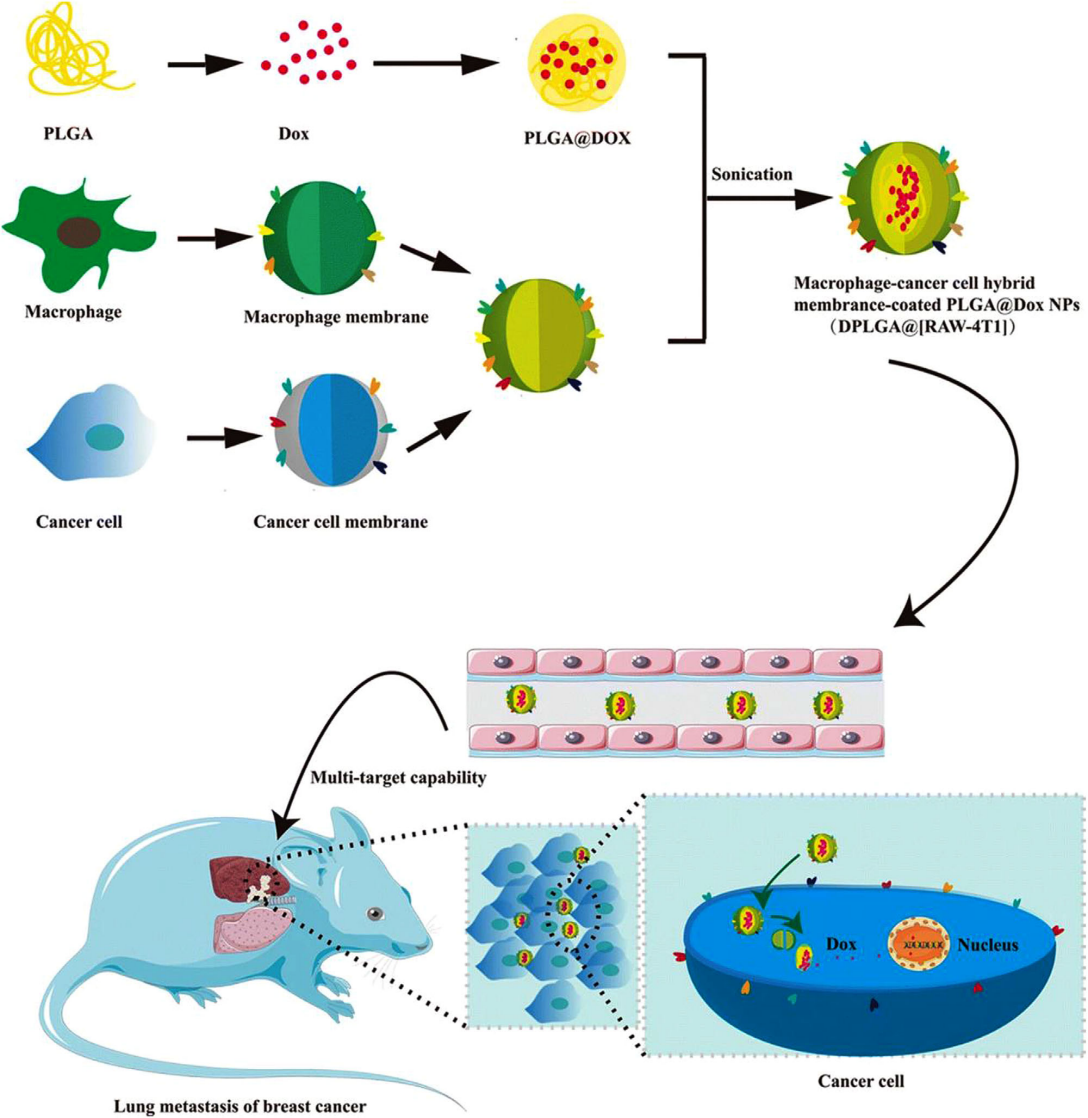
Schematic illustration of RAW‐4T1 hybrid membrane‐based drug delivery system applied for treating lung metastases of breast tumor. The synthetic RAW‐4T1 hybrid membrane coated (DOX‐loaded PLGA nanoparticles (DPLGA@[RAW‐4T1] NPs) enhanced tumor‐targeted delivery of DOX due to their multi‐target capability, thereby elicited significant inhibition of lung metastases in breast tumor. Reproduced with permission.^[^ ^241^ ^]^ Copyright 2020, Springer Nature

Recently, the emerging membrane‐hybridization technologies have attracted considerable attention, because the hybrid membranes possess functions of different cell membranes.^[^ ^241^ ^]^ Accordingly, Gong et al. synthesized DOX‐loaded RAW 264.7‐4T1 hybrid biomimetic membrane camouflaged‐PLGA NPs (DPLGA@[RAW‐4T1] NPs), which possessed multi‐tumor‐targeting capability and metastasis‐targeting capability, because the overexpressed *α*_4_ *β*_1_ integrins on the macrophage membranes can interact with 4T1 cell overexpressed‐VCAM‐1, and the 4T1 membrane could also target 4T1 tumor cells (Figure 12).^[^ ^241^ ^]^ The hybrid cell membrane dual‐delivery system triggered robust anti‐metastasis efficacy (approximately 88.9%), which suggests the application of hybrid cell membrane‐disguised nanoplatforms for specific tumor metastasis‐targeting therapy.

### 4.3 Macrophage‐derived extracellular vesicles

Recently, EVs have been gained extensive attentions and exploited as drug carriers for anti‐tumor therapy.^[^ ^245^ ^]^ Based on their subcellular origin and size, EVs can be divided into three classes: (1) Exosomes (Exos, 30–150 nm in diameter), which are formed by fusing the multivesicular bodies (MVBs) with plasma membrane, (2) microvesicles (MiVs, also denoted as microparticles or ectosome, 100–1000 nm), which are directly shed or budded from cell membranes, (3) apoptotic bodies (ApB, 500 nm–5 μm), which are released by dying/apoptotic cells.^[^ ^30^, ^33^ ^]^ The first two classes are widely applied for anti‐tumor therapy. Generally, EVs carry integrins, proteins, nucleic acids, and lipids from their parental cells, and have specific biological functions in line with their sourced cells.^[^ ^30^ ^]^ For example, macrophage‐derived exosomes exhibit membrane surface properties similar to those of macrophages, and own tumor tropism capability.^[^ ^33^, ^246^, ^247^ ^]^ Moreover, accompanied by changes in macrophage phenotype, M1‐like macrophage‐derived EVs show anti‐tumor functions, and can promote pro‐inflammatory immune responses.^[^ ^30^, ^247^ ^]^ In contrast, M2‐like macrophage‐derived EVs display pro‐tumor functions and can elicit anti‐inflammatory response to promote tumor migration, invasion, and metastasis.^[^ ^30^, ^248^ ^]^ Therefore, macrophage or M1‐like macrophage‐derived Exos and MiVs are extensively applied to deliver anti‐tumor agents and improve anti‐tumor efficacy.

**FIGURE 13:**
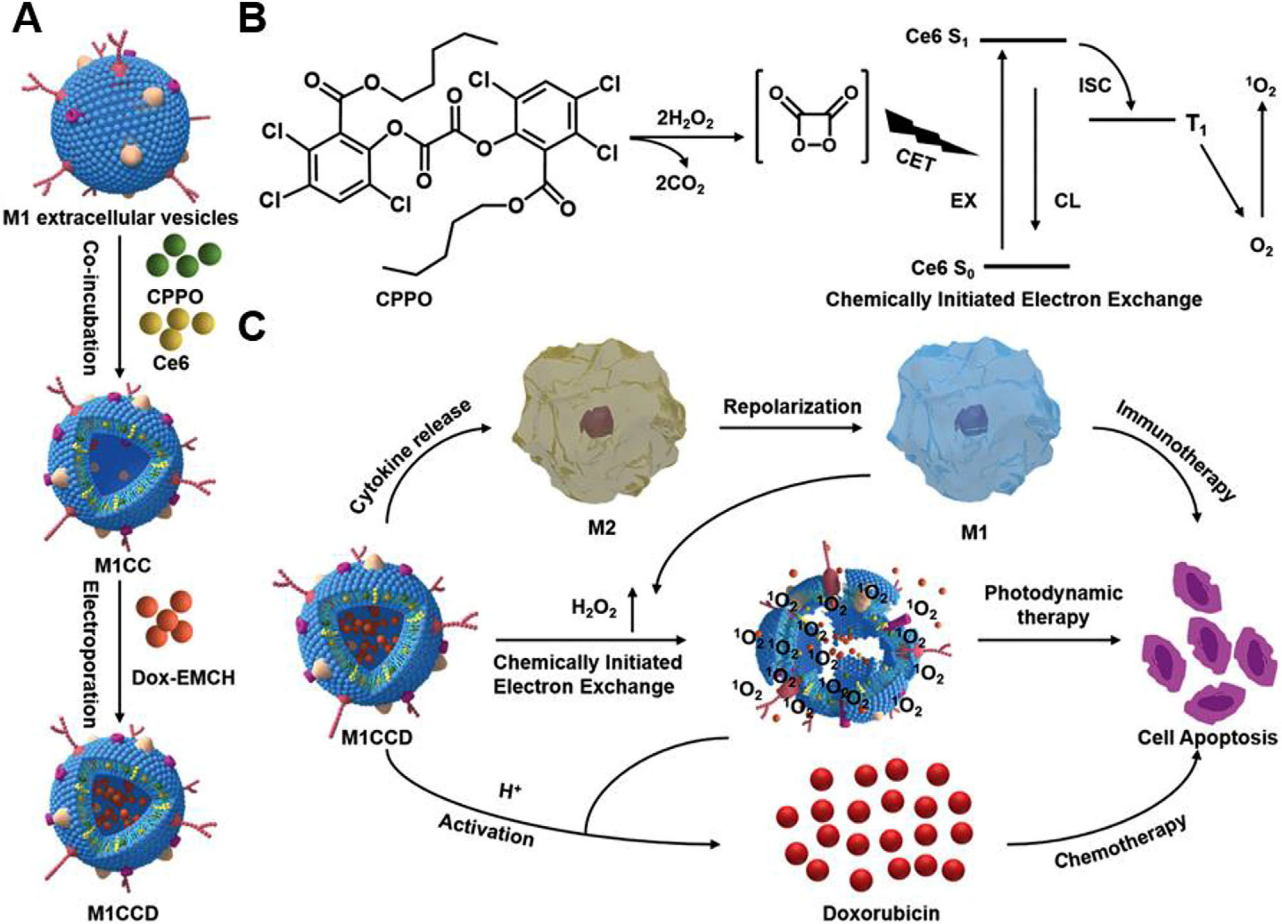
Schematic illustration of utilizing the M1CCD for trimodal anti‐tumor therapy. (A) Fabrication of M1CCD. (B) Mechanism of chemically triggered luminescence and the singlet oxygen generation. (C) The mechanism of synergistic tumor inhibition of M1CCD. Reproduced with permission.^[^ ^247^ ^]^ Copyright 2021, John Wiley & Sons

Generally, M1‐derived EVs possess more advantages than those of unstimulated M0 like macrophages, because they inherit anti‐tumor functions of M1‐like macrophages. Ding et al. engineered a type of self‐activatable M1‐like macrophage‐derived EVs (M1 EVs), which were loaded with bis[2,4,5‐trichloro‐6‐(pentyloxycarbonyl) phenyl] oxalate (CPPO), chlorin e6 (Ce6), and prodrug aldoxorubicin (DOX‐EMCH), to treat 4T1 tumor (Figure 13).^[^ ^247^ ^]^ The obtained CPPO, Ce6, and DOX‐EMCH‐loaded EVs (M1CCD) could efficiently target tumor cells due to the natural tumor‐homing capability of M1 EVs. More importantly, the M1 EVs also remarkably reprogramed pro‐tumor M2‐like TAMs to anti‐tumor M1 phenotype, and generated large amounts of H_2_O_2_, which can react with CPPO to generate chemical energy to activate Ce6 for deep PDT. Interestingly, PDT‐induced‐^1^O_2_ facilitated membrane rupture to release DOX‐EMCH, resulting in deep penetration of drugs into the hypoxic area of tumor. Therefore, the M1 Ev‐based systems display great capability to inhibit tumor progression due to the combined effect of various components.

#### 4.3.1 Macrophage‐derived exosomes

To further improve tumor‐targeting capability of Exo, surface modification has been extensively used. Li et al. prepared macrophage‐derived Exos‐coated poly(lactic‐*co*‐glycolic acid) nanoplatform for tumor‐targeted delivery of DOX, and then utilized a mesenchymal‐epithelial transition factor (c‐Met)‐targeting peptide to modify the Exos surface, because c‐Met is overexpressed by triple‐negative breast cancer cells.^[^ ^246^ ^]^ The resultant nanoparticles showed robust tumor‐targeted delivery efficacy of DOX due to the dual tumor‐homing capabilities of macrophage‐derived Exos and c‐Met‐targeting peptide, which resulted in remarkable tumor cell apoptosis and regression of tumor growth. Wang et al. fabricated M1‐like macrophage‐derived exosome‐based PTX delivery system, which could induce potent anti‐tumor efficacy of chemotherapy, because M1‐Exos activated classical M1‐associated NF‐*κ*B‐mediated pro‐inflammatory response in the TME.^[^ ^249^ ^]^

#### 4.3.2 Macrophage‐derived microvesicles

Compared with the extensively developed Exo‐based drug delivery system, MiVs are overlooked. Guo et al. found that M1‐like macrophage‐derived MiVs own superior advantageous features for tumor‐targeting delivery of chemotherapeutic drugs compared with Exos.^[^ ^33^ ^]^ One reason is that MiVs are directly budded from the cell membrane, largely inherit the parental cell membrane properties, and exhibit CCL2/CCR2‐mediated tumor‐homing function. While Exos are derived through fusing MVBs with membrane, and may possess high level proteins from endosomal membrane. The authors demonstrated a novel mechanism that the M1‐like macrophage‐derived MiV could directly deliver DOX into nucleus via SNARE‐mediated membrane fusion manner, which can avoid endocytic degradation. In addition, Wei et al. developed mannose‐modified macrophage‐derived microparticles (Man‐MPs, in which MPs were also denoted as microvesicles (MiVs)) for targeting M2‐like TAMs by delivery of TAMs‐repolarization drug metformin (Met@Man‐MPs), which efficiently reversed tumor‐supportive TAMs into anti‐tumor M1 phenotype.^[^ ^250^ ^]^ Because macrophages express MMPs, Met@ManMPs promoted tumor ECM degradation and significantly facilitated tumor penetration and tumor accumulation of anti‐PD‐1 antibody, which elicited CD8^+^ T cell immunity and boosted anti‐PD‐1 immunotherapy. More importantly, whether MiVs were derived from RAW264.7 cells, BMDMs, human monocytic THP‐1‐derived macrophages or human peripheral blood monocyte‐derived macrophages (MDMs), Met@Man‐MPs exhibited similar functions of reprograming M2‐like macrophages into M1‐like ones, and expressed MMPs, which suggests that the performances of MiVs from different types of macrophages are universal.

#### 4.3.3 Macrophage‐derived nanovesicles

**FIGURE 14:**
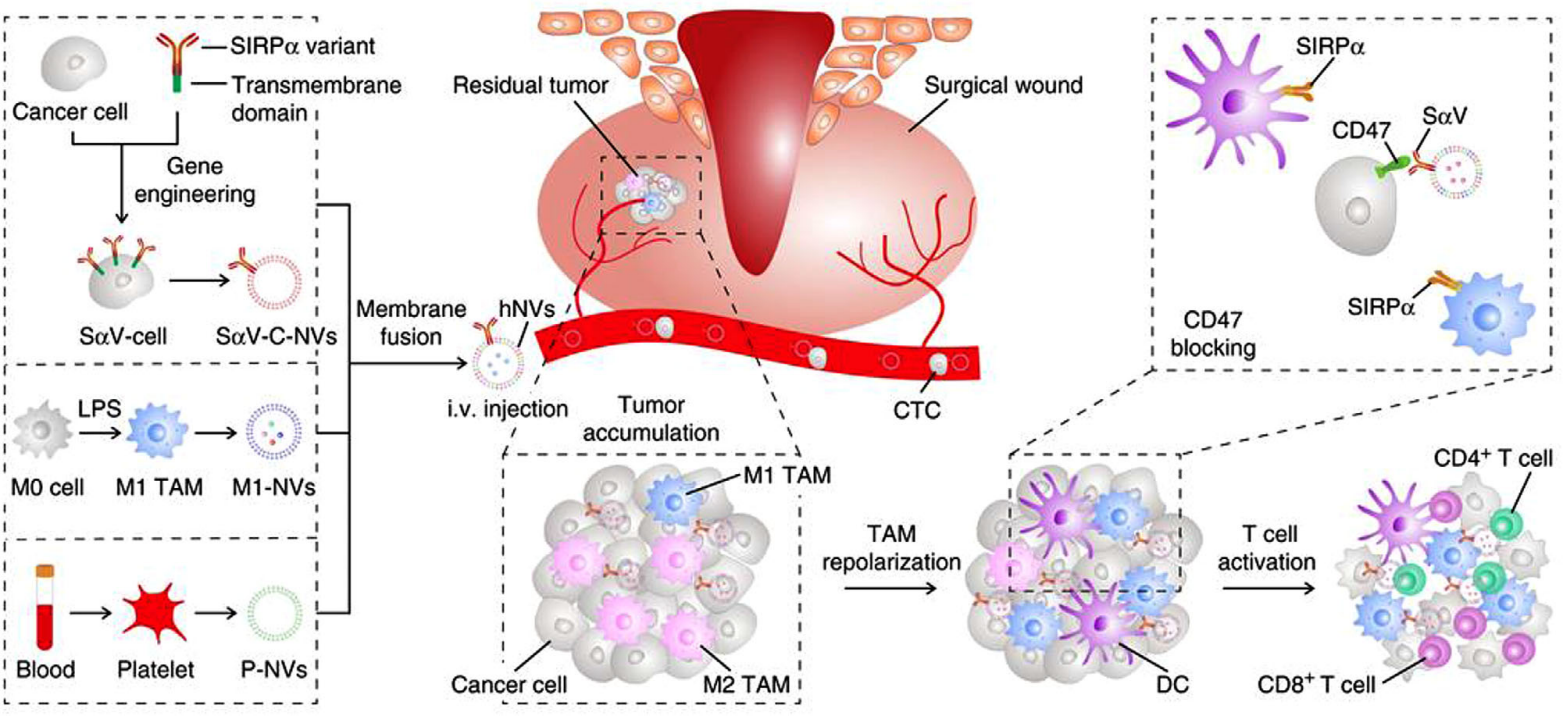
Schematic illustration of isolating S*α*V‐C‐NVs, M1‐NVs, and P‐NVs for producing the hNVs to inhibit tumor metastasis and recurrence. The hNVs could target the post‐surgical tumor site, recognize and interact with CTCs, and block CD47‐SIRP*α* anti‐phagocytic checkpoint, thereby enhancing tumor phagocytosis of macrophages, promoting M2‐to‐M1 repolarization, and potentiating tumor‐specific T cell immunity to result in robust suppression of tumor metastasis and recurrence. Reproduced with permission.^[^ ^252^ ^]^ Copyright 2020, Springer Nature

The challenge of EVs (Exos or MiVs)‐based drug delivery is that cells usually secrete a small quantity of EVs, which is insufficient for anti‐tumor drug delivery.^[^ ^251^, ^252^ ^]^ Alternatively, the cellular nanovesicles (NVs) have been demonstrated to be a nice substitute, because they can be highly yielded by serial sonication and extrusion of cells.^[^ ^127^, ^252^ ^]^ Recently, Rao et al. engineered hybrid cellular membrane nanovesicles, which were fused by three different cell derived NVs including platelet‐derived NVs (P‐NVs), M1‐like macrophage‐derived NVs (M1‐NVs), and cancer cell‐derived NVs with overexpression of high‐affinity SIRP*α* variants (S*α*V‐C‐NVs) (Figure 14).^[^ ^252^ ^]^ Since P‐NVs can interact with circulating tumor cells (CTCs), M1‐NVs can reprogram tumor‐supportive TAMs into anti‐tumor M1 phenotype, and blocking CD47‐SIRP*α* axis can promote tumor‐phagocytosis of macrophages, the resultant hNVs inherit the properties of source cells. They could efficiently target and accumulate into surgical wound sites, recognize and interact with CTCs in the blood, promote M2‐to‐M1 repolarization, and elevate tumor‐engulfment of macrophages via interdicting CD47‐SIRP*α* phagocytic checkpoint. These roles synergistically resulted in robust resistance to tumor recurrence and metastasis.

In summary, although macrophages and macrophage‐derived components (i.e., macrophage membrane and macrophage‐derived EVs) are able to home to primary and metastatic tumor, there are certain differences between them. For macrophage membrane and macrophage‐derived‐EVs, they usually loose some functional membrane proteins and would inherit inadequate tumor tropism from macrophages.^[^ ^33^, ^36^ ^]^ The living macrophages can maximally maintain their components and functions. They can only load sub‐lethal dose of drugs to maintain their survival and activity, while macrophage membranes are able to encapsulate high dose of drugs. Moreover, once the drugs are internalized by macrophages, they are subject to degradation in macrophage phagosomes and result in insufficient tumor‐targeted drug delivery.^[^ ^29^, ^36^ ^]^ Furthermore, unlike the cell membrane, both M1‐like macrophage and M1‐like macrophage‐derived EVs retain anti‐tumor capability. However, adoptive administration of M1‐like macrophages usually face challenge of maintaining their anti‐tumor phenotype. M1‐like macrophage‐derived EVs can inherit the characteristic of M1‐like macrophages such as pro‐inflammatory and antigen presentation capability.^[^ ^30^, ^187^ ^]^

## 5 CONCLUSIONS AND PERSPECTIVES

TAMs are the most abundant immunosuppressive cells within the TME. Modulation of TAMs through strategies of inhibiting circulating TAMs precursors, depleting TAMs, and repolarizing TAMs has shown great successes in improvement of anti‐tumor efficacy. Due to M2‐like TAMs have natural ability of inhibiting T cell activity and M1 phenotype macrophages display potent antigen presentation ability, the aforementioned strategies hold great potential in improving tumor‐specific T cell‐based adaptive immunity and manipulating macrophage‐mediated phagocytosis of tumors. Furthermore, enhancing macrophage‐mediated engulfment of tumor cells and blocking macrophage‐mediated efferocytosis are also feasible for preventing tumor cell evasion. Blocking macrophage‐mediated efferocytosis could induce ICD of un‐eliminated dying/apoptotic tumor cells to release DAMPs and enhance adaptive immunity. Macrophage‐based drug delivery is also very attractive for primary and metastatic tumor‐targeting due to the capability of crossing many complex and insurmountable biological barriers, such as, the BBB, which is very challenging for delivering nanoparticles.

Nanoscience and nanotechnology have shown promise in macrophage‐based tumor immunotherapy. Nanoparticles can be finely tuned to serve as promising carriers of TAMs‐modulators by surface modification. They can be fabricated with appropriate shape, size, surface charge, and targeting ligands. These advantages enhance tumor‐targeted delivery efficacy, prolong blood circulation, maintain regulative effect of conventional TAMs‐modulators. In addition, several types of nanoparticles are able to directly regulate TAMs functions, which can further expand the window for TAMs‐targeted tumor immunotherapy. Although nanotechnology is promising for improve TAMs‐based tumor immunotherapy, various limitations still exist. First, the complex TME consists of multiple biological barriers such as dense ECM, high IFP, heterogeneous blood supply, tumor stroma, etc., which are serious obstacles for nanoparticles to penetrate into the deep tumor area. Therefore, nanoparticles should be rationally designed to simultaneously overcome multiple biological barriers. Second, many simple nanoparticle‐based carriers cannot release drugs in a controlled manner. Intelligent nanocarriers with spatio‐temporal controlled drug release kinetics should be designed to further improve drug delivery efficiency and utilization, as well as, overall TAMs‐regulating effect. Third, very few nanoparticles can cross the BBB, which is an important obstacle in regulating the function of macrophages in the glioma. Therefore, it's necessary to design smart nanoparticles or assist with other methods, such as, the noninvasive focused ultrasound (US) technique,^[^ ^253^, ^254^, ^255^, ^256^ ^]^ to cross the BBB for improving the efficacy of TAMs‐based immunotherapy of glioma.

Despite of the great success in engineering macrophage‐based immunotherapy for tumor treatment, various challenges remain to be solved. First, inhibiting circulated precursors of TAMs or directly depleting them would indiscriminately ablate all types of macrophages in the tumor environment, which could weak their intrinsic capability of tumor‐phagocytosis and antigen presentation, and disturb in vivo homeostasis of macrophages. How to selectively inhibit or deplete TAMs precursors is very challenging. Thus, the alternative strategies such as TAMs‐repolarization and modulation of macrophage‐mediated phagocytosis could be good choices for macrophage‐based immunotherapy. Second, it is well known that macrophages are highly plastic and easily altered upon stimulation, the obtained M1‐like macrophages would rapidly turn into immunosuppressive M2 macrophages under the stimulation. For example, M1‐like macrophages metabolize through glycolysis to produce lactic acid, which would turn them back into M2 phenotype again. The adoptively transferred macrophages and macrophage‐based drug carriers are usually subjected to M2‐inducing cues in their surrounding environment. Therefore, how to induce durable anti‐tumor M1 macrophages is another challenge for macrophage‐based immunotherapy. Third, among phagocytotic checkpoints, the PD‐L1‐PD‐1 axis is also a critical and popular adaptive checkpoint. However, it's very challenging to completely distinguish the inhibition of PD‐1‐PD‐L1 axis in T cells and macrophages. Their blockers should be selectively delivered to macrophages when tumor‐phagocytosis of macrophages was considered only. More importantly, whether the same regulator could respond diversely in different types of cells remains to be elucidated.

For inhibiting macrophage‐mediated efferocytosis, it is also very challenging to completely block this process. On the one hand, MerTK signaling is also involved in stimulation of T cells, and inhibiting MerTK signaling could induce opposite effects between functions of macrophages and T cells. Restimulating MerTK‐positive cells could elevate the central memory pool, and MerTK inhibition could abrogate this process, induce short‐term responses of effector T cells instead of long‐term T cell memory, and fail to prevent tumor recurrence in a long term. On the other hand, MerTK is also expressed on tumor cells and DCs, which play important roles in promoting tumor progression and inducing DCs‐mediated immunosuppressive condition. Inhibiting MerTK would also affect the functions of DCs. Therefore, personalized macrophage‐targeting strategies should be developed for MerTK‐targeting.

For engineering macrophages as drug delivery vehicles, the first challenge would be the low encapsulation of drugs, nanoparticles and their composites into the macrophages, because high payloads could be highly toxic to cells. Therefore, it is necessary to develop intelligent designs to reduce payloads toxicity with an improved drug‐loading efficiency. The second challenge lies in engineering macrophages as “Trojan Horses”, because the living macrophages (M0‐ or M1‐ like macrophages) are subjected to environmental cues and could turn into pro‐tumor M2 phenotype, whether they phagocytose payloads or carry the BPs. Polarization of macrophages into M2 phenotype could lead to the failure of anti‐tumor therapy and exacerbation of tumor progression. Therefore, it is urgent to develop intelligent macrophage‐based drug delivery systems to protect or maintain M1 phenotypes in a long term. Since macrophage‐derived EVs are originated from donor cell membrane, which inherit part of the cell membrane function and compromise their tumor‐targeting capability. Therefore, further genetical engineering or surface modifying of macrophage or macrophage‐derived EVs with specific tumor‐targeting ligands is necessary to improve their tumor‐targeting efficiency.

It should be noted that the tumor‐immunity was usually manipulated by multiple cascade steps, including: (1) Release of tumor antigens, (2) APC‐mediate antigen presentation in the secondary lymphoid organs, (3) migration of CTLs cells to the primary and metastatic tumor sites to kill tumors. It is not possible to completely kill tumor through macrophage‐based immunotherapy. Therefore, combining macrophage‐based anti‐tumor therapy with other immunotherapy methods to boost the complete tumor‐immunity or designing multifunctional platforms would be very promising to maximize the anti‐tumor immune efficacy.

## CONFLICT OF INTEREST

The authors declare no conflict of interest.
